# Metabolic Blockade-Based Genome Mining of *Malbranchea circinata* SDU050: Discovery of Diverse Secondary Metabolites

**DOI:** 10.3390/md23010050

**Published:** 2025-01-20

**Authors:** Hu Yang, Xiaowei Luo, Zhuo Shang, Kunlong Li, Jian Cai, Yingying Chen, Longchao Xin, Jianhua Ju

**Affiliations:** 1 Key Laboratory of Chemical Biology (Ministry of Education), Shandong Basic Science Research Center (Pharmacy), School of Pharmaceutical Sciences, Cheeloo College of Medicine, Shandong University, Jinan 250012, China; 202120941@mail.sdu.edu.cn (H.Y.); zshang@sdu.edu.cn (Z.S.); lklong@sdu.edu.cn (K.L.); xinlongchao@foxmail.com (L.X.); 2 Guangxi Key Laboratory of Marine Drugs, University Engineering Research Center of High-Efficient Utilization of Marine Traditional Chinese Medicine Resources, Institute of Marine Drugs, Guangxi University of Chinese Medicine, Nanning 530200, China; luoxiaowei1991@126.com; 3 CAS Key Laboratory of Tropical Marine Bio-Resources and Ecology, South China Sea Institute of Oceanology, Chinese Academy of Sciences, Guangzhou 510301, China; caijian@scsio.ac.cn (J.C.); chenyingying7788@163.com (Y.C.)

**Keywords:** secondary metabolites, fungi, deep-sea sediment, metabolic blockade, cytochalasin

## Abstract

*Malbranchea circinata* SDU050, a fungus derived from deep-sea sediment, is a prolific producer of diverse secondary metabolites. Genome sequencing revealed the presence of at least 69 biosynthetic gene clusters (BGCs), including 30 encoding type I polyketide synthases (PKSs). This study reports the isolation and identification of four classes of secondary metabolites from wild-type *M. circinata* SDU050, alongside five additional metabolite classes, including three novel cytochalasins (**7**–**9**), obtained from a mutant strain through the metabolic blockade strategy. Furthermore, bioinformatic analysis of the BGC associated with the isocoumarin sclerin (**1**) enabled the deduction of its biosynthetic pathway based on gene function predictions. Bioactivity assays demonstrated that sclerin (**1**) and (−)-mycousnine (**10**) exhibited weak antibacterial activity against Gram-positive bacteria such as *Staphylococcus aureus*, methicillin-resistant *Staphylococcus aureus* (MRSA), and *Bacillus subtilis*. These findings underscore the chemical diversity and biosynthetic potential of *M. circinata* SDU050 and highlight an effective strategy for exploring marine fungal metabolites.

## 1. Introduction

The deep sea is characterized by high pressure, high salinity, low temperature, and perpetual darkness, making it one of the most extreme environments on Earth [1,2]. These harsh conditions have driven the evolution of unique fungi capable of producing diverse secondary metabolites, which aid their survival and adaptation [3,4]. Many of these secondary metabolites, such as lithocarpins [5], arthpyrones [6], and spirograterpenes [7], hold significant potential as prototypes for drug development. However, the frequent rediscovery of known metabolites and the difficulty in accessing cryptic metabolites under standard laboratory culture conditions pose challenges to the discovery of novel compounds [8]. Recent advances in genome mining and metabolic engineering strategies, including one-strain-many-compounds (OSMAC) [9], ribosome engineering [10], gene overexpression [11], heterologous expression [12], metabolic shunting [13], self-resistant gene-directed genome mining [14], and a metabolic blockade strategy developed by our group [15,16], have enabled the activation of cryptic biosynthetic gene clusters (BGCs). These approaches have paved the way for uncovering novel secondary metabolites with diverse biological activities from deep-sea microorganisms.

**Figure 1:**
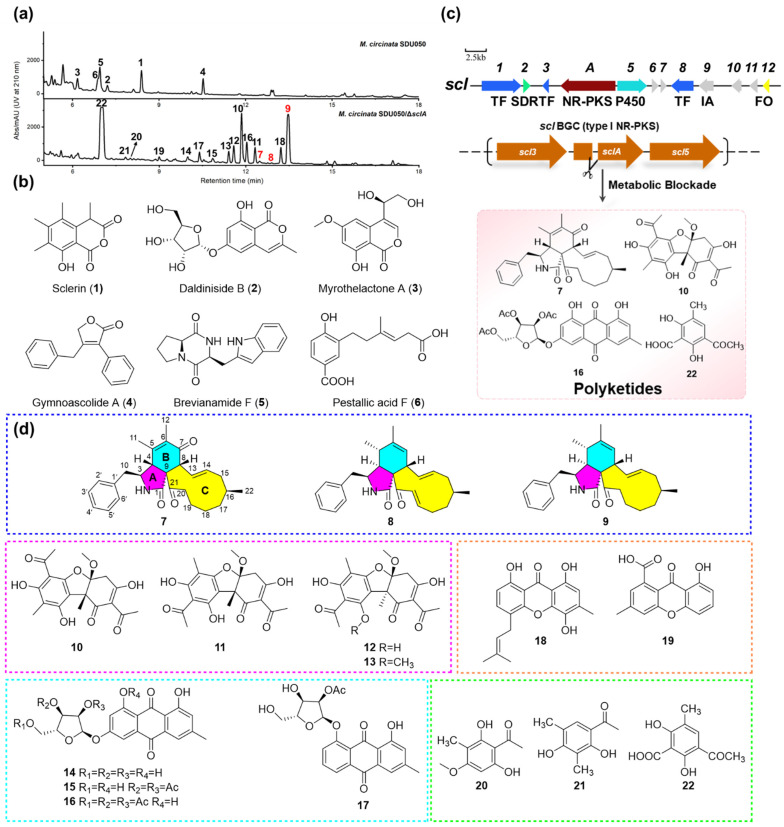
(**a**) HPLC analyses of crude extracts from the wild-type and Δ*sclA* mutant strains fermented in PDB media. The number on the peaks corresponds to the products shown in (**b**,**c**). (**b**) Structures of compounds **1**–**6** isolated from the wild-type *M. circinata* SDU050. (**c**) Organization of *scl* BGC and the process of metabolic blockade strategy for *M. circinata* SDU050. TF: transcription factor; SDR: short-chain dehydrogenase; NR-PKS: non-reducing polyketide synthase; P450: cytochrome P450 monooxygenase; IA: isomerase; FO: FAD dependent oxidase. (**d**) Structures of compounds **7**–**22** isolated from *M. circinata* SDU050/Δ*sclA* mutant strain.

In the search for new antibacterial secondary metabolites from marine fungi, we identified a deep-sea sediment-derived fungus *Malbranchea circinata* SDU050 through activity-guided screening. The genus *Malbranchea* is known for producing structurally diverse and biologically active secondary metabolites, including pyrrole alkaloids with anticancer properties, indole terpenoid alkaloids with vasorelaxant and calmodulin inhibitor activities, and polyketides with antidiabetic potential [17,18,19]. After the scale-up fermentation of *M. circinata* SDU050 in potato dextrose broth (PDB), we isolated and identified six compounds, including three isocoumarins (**1**–**3**), a butyrolactone derivative (**4**), a dipeptide (**5**), and a hydroxybenzoic acid derivative (**6**). The genome sequencing and bioinformatic analysis of *M. circinata* SDU050 revealed 69 putative BGCs, including 30 encoding type I polyketide synthases (T1PKSs). However, most of the metabolites predicted from these BGCs were absent in the extract of *M. circinata* SDU050, suggesting that many of these clusters are silent or minimally expressed under standard culture conditions. To explore the biosynthetic potential of this strain, a metabolic blockade strategy was employed to activate or enhance the production of additional polyketides by inactivating the core PKS gene responsible for the biosynthesis of sclerin (**1**), the predominant metabolite produced by the strain. This approach successfully led to the isolation and identification of three new cytochalasins (**7**–**9**), along with four usnic acid derivatives (**10**–**13**), four glycosylated anthraquinones (**14**–**17**), two xanthones (**18**–**19**), and three acetophenone derivatives (**20**–**22**). Compounds **1** and **10**–**13** were evaluated for their antibacterial activity and all isolates were evaluated for cytotoxic activity. This paper presents the isolation, structure elucidation, and bioactivity of these secondary metabolites as well as the identification of the PKS responsible for the biosynthesis of sclerin (**1**) by Crispr-based gene inactivation (Figure 1 and Figure S2).

## 2. Results

### 2.1. Metabolite Investigation of the Strain M. circinata SDU050

To investigate the potential of *M. circinata* SDU050 for producing bioactive secondary metabolites, a 6L fermentation of the wild-type *M. circinata* SDU050 was carried out using PDB medium followed by EtOAc extraction and high-performance liquid chromatography (HPLC) analysis (Figure 1a). Next, the EtOAc extract of broth and the acetone extract of mycelia were combined and subjected to multiple rounds of chromatography, yielding compounds **1**–**6**. Through an NMR spectral data comparison with the literature, compound **1** was identified as sclerin, a toxin molecule featuring an isocoumarin scaffold [20]. Compounds **2** and **3** were identified as daldiniside B [21] and myrothelactone A [22], respectively, which also belong to the isocoumarin family. Accordingly, compounds **4**–**6** were characterized as butenolide gymnoascolide A (**4**) [23], dipeptide brevianamide F (**5**) [24], and hydroxybenzoic acid derivative pestallic acid F (**6**) [25]. The structures of **1**–**6** are illustrated in Figure 1b.

### 2.2. Genome Sequencing and Bioinformatic Analysis of M. circinata SDU050

To identify secondary metabolite BGCs, the whole-genome sequence of *M. circinata* SDU050 was analyzed using antiSMASH 7.0 [26]. The bioinformatic analysis [27] indicated that the genome encodes 69 BGCs, comprising 30 type I polyketide synthase (T1PKS), 5 nonribosomal peptide synthase (NRPS), 10 NRPS-like, 12 ribosomally synthesized and post-translationally modified peptide (RiPP), 5 terpene synthase (TS), 3 PKS/NRPS hybrid, 1 PKS/NRPS-like hybrid, and 3 indole synthase clusters (Table S1). These findings, especially the large number of PKS gene clusters, suggest that *M. circinata* SDU050 possesses the potential to produce at least 69 structurally distinct secondary metabolites, far exceeding the number of predominant metabolites detected in the PDB broth. To further explore the chemical diversity of polyketides in *M. circinata* SDU050, an efficient metabolic blockade strategy was applied. This approach involves disrupting key biosynthetic pathways, thereby diverting precursors and cellular energy to alternative BGCs, which activates or enhances the production of previously undetected metabolites.

### 2.3. Metabolic Blockade-Driven Genome Mining Unveils a Wealth of Polyketides

To apply the metabolic blockade approach, we first aimed to locate and disrupt the biosynthesis of the predominant metabolite, sclerin (**1**), produced by *M. circinata* SDU050. A local BLAST homology search was conducted against all PKS clusters in the genome using the PKS TerA sequence as a query, which is involved in 6-hydroxymellein biosynthesis [28]. This search identified a highly homologous NR-PKS gene, namely, *sclA*, located in contig **1** (Table S2). To confirm whether *sclA* is involved in the biosynthesis of **1**, the gene was deleted using the CRISPR/Cas9 system, generating the mutant strain Δ*sclA*. HPLC analysis confirmed that the Δ*sclA* mutant lost the ability to produce **1** completely in PDB media, establishing that NR-PKS SclA is responsible for its biosynthesis in *M. circinata* SDU050 (Figure 1c and Figure S2). Instead, a series of previously unobserved peaks was detected, indicating that the metabolic blockade strategy successfully activated or increased the production of previously undetected secondary metabolites (Figure 1a). A subsequent 20 L fermentation of the Δ*sclA* mutant strain resulted in the isolation and identification of 16 polyketides, classified into five PKS families: cytochalasins (**7**–**9**), usnic acids (**10**–**13**), glycosylated anthraquinones (**14**–**17**), xanthones (**18**–**19**), and acetophenone derivatives (**20**–**22**). Notably, three cytochalasins (**7**–**9**) were identified for the first time (Figure 1d).

**Figure 2:**
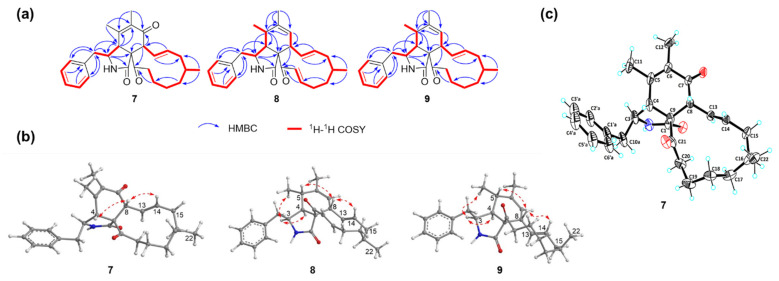
(**a**) ^1^H-^1^H COSY and key HMBC correlations of compounds **7**–**9**. (**b**) Key NOESY correlations of compounds **7**–**9**. (**c**) X-ray crystallographic structure of compound **7**.

Malcirchalasin A (**7**) was isolated as a colorless solid. Its molecular formula was deduced as C_27_H_33_NO_3_ based on the molecular ion in the HRESIMS ([M+H]^+^, *m*/*z* 420.2539; calcd. for C_27_H_34_NO_3_, 420.2533), indicating twelve degrees of unsaturation. The ^1^H NMR spectral data of **7** showed three methyls, a phenyl ring, and two olefin protons at *δ*_H_ 6.36 (dd, *J* = 15.6, 9.9 Hz) and 5.50 (ddd, *J* = 15.0, 10.4, 4.1 Hz). The ^13^C NMR spectral data of **7** displayed 27 carbon signals, including three methyls, six methylenes, eleven methines, and seven nonprotonated carbons including three carbonyl carbons (Table S7 and Figures S4–S8). The structure of **7** was established by comprehensive analysis of its 2D NMR spectral data (Figure 2a and Figures S9–S11). Based on the ^1^H-^1^H COSY correlations of H-4/H-3/H_2_-10 together with the HMBC correlations of H-3/C-1 and C-9; H-4/C-3, C-5 and C-6; H-8/C-7 and C-9; H_3_-11/C-4 and C-6; and H_3_-12/C-5 and C-7, the presence of a penhydroisoindolone moiety (rings A and B) was determined, which involve a disubstituted *α*, *β*-unsaturated ketone in the B ring. The spin systems of H-2′/H-3′/H-4′/H-5′/H-6′ and the HMBC correlations of H-2′ and H-6′/C-10 and H_2_-10/C-3 and C-4 suggested that the benzyl group was attached to C-3. Furthermore, the eleven-membered ring C was correlated by the ^1^H-^1^H COSY correlations of H-8/H-13/H-14/H_2_-15/H-16/H_2_-17/H_2_-18/H_2_-19/H_2_-20 and the HMBC correlations of H-14/C-8; H-13/C-15; H_3_-22/C-15 and C-17; H_2_-17/C-19 and H-8; and H_2_-19/C-21. Thus, the two-dimensional structure of **7** was deduced to be a 5/6/11 tricyclic ring system, which was determined as a new cytochalasin.

**Figure 3:**
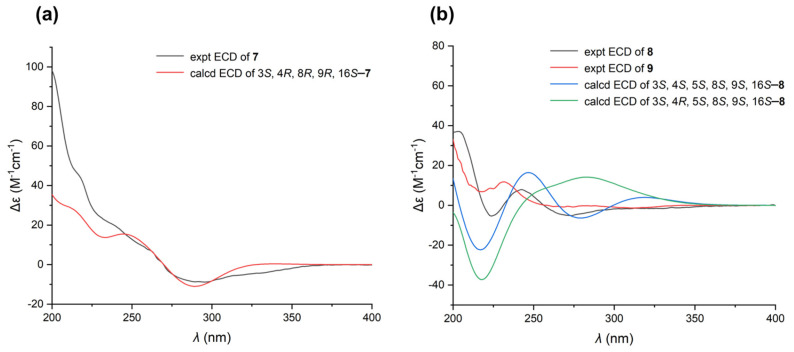
(**a**) Experimental ECD and calculated ECD spectra of **7**. (**b**) Experimental ECD of **8** and **9** and calculated ECD spectra of **8**.

The NOESY correlations (Figure 2b and Figure S12) of H-4/H-8 indicated that these protons were on the same plane in **7**, as depicted in Figure 2b. However, the relative configuration of H-3 and H_3_-22 could not be determined with certainty due to the absence of key NOE correlations. Fortunately, we successfully obtained the crystal of **7** for single-crystal X-ray diffraction analysis (CCDC 2413422) (Figure 2c and Figure S1, Table S8). Although Cu radiation was employed for the analysis, only the relative configuration of **7** was determined because of the relatively low quality of the crystal with the Flack parameter of 0.3(3) for **7**, which is not lower enough for absolute configuration determination. The crystal structure of **7** revealed a 3S*, 4R*, 8R*, 9R*, 16S* configuration and demonstrated that H-4 and H-8 were on the same plane, which was consistent with the NOE correlations. Given that the NRPS for loading the phenylalanine lacks the epimerization (E) domain (Figure S3), we tentatively assigned a 3*S* absolute configuration in **7**. Furthermore, the ECD calculations were carried out using time-dependent density–functional theory (TDDFT) at the B3LYP/6–31+g(d,p) level [29]. The ECD calculation of (3*S*, 4*R*, 8*R*, 9*R*, 16*S*)-**7** yielded an almost identical Cotton effect to the experimental one. Thus, the absolute configuration of **7** was tentatively assigned as 3*S*, 4*R*, 8*R*, 9*R*, 16*S* (Figure 3a and Figure S29).

Malcirchalasin B (**8**) was isolated as a white solid. The molecular formula was determined as C_27_H_33_NO_2_, based on the HRESIMS data ([M+H]^+^, *m*/*z* 404.2590; calcd. for C_27_H_34_NO_2_, 404.2584), indicating twelve degrees of unsaturation. A detailed analysis of the ^1^H and ^13^C NMR spectral data indicated that compound **8** could be a homolog to compound **7** with three methyls, four methylenes, fifteen methines, and five nonprotonated carbons including two carbonyl carbons (Table S7 and Figures S13–S16). Comparing the ^1^H and ^13^C NMR spectral data of **7** and **8** indicated that two more pairs of olefin protons at *δ*_H_ 6.29 (ddd, *J* = 16.2, 10.8, 5.8 Hz) and 6.82 (d, *J* = 16.2 Hz) and carbons at *δ*_C_ 147.3 and 132.8 in **8** replaced two corresponding pairs of alkyl protons and carbons in **7**. The COSY correlations of H_2_-18/H-19/H-20 and the HMBC correlations of H_2_-17/C-19 and H-19, H-20/C-21 further confirmed the location of the double bond (Figure 2a). In addition, another difference in the B ring, i.e., that one carbonyl group is missing in **8** compared with **7,** was established through a comprehensive analysis of its 2D NMR spectral data (Figures S17–S19). Based on the ^1^H-^1^H COSY correlations of H_3_-11/H-5/H-4/H-3/H_2_-10 and H-7/H-8 together with the HMBC correlations of H-5/C-3, C-4, C-6 and C-9; H_3_-12/C-5, C-6 and C-7; and H-7/C-5 and C-8, the precise structure of the B ring was determined (Figure 2a). Thus, the planar structure of **8** was determined.

The relative configuration of **8** was elucidated by analyzing the NOESY correlations (Figure 2b and Figure S20). The cross-peaks of H-3/H-4/H_3_-11 suggested that these three protons were at the same face. In addition, the correlations of H-5/H-8 indicated that these protons were at the opposite face. Considering that compounds **7**–**9** are all derived from the same biosynthetic pathway, the absolute configuration of C-16 in **8** can be tentatively assigned as *S*, the same as in **7**. In order to determine the stereochemistry of other chiral centers, the calculated ECD spectra for **8** were also performed and the absolute configuration of **8** was assigned as 3*S*, 4*S*, 5*S*, 8*S*, 9*S*, 16*S* (Figure 3b and Figure S30).

Malcirchalasin C (**9**) was isolated as a white paste. Its molecular formula was determined as C_27_H_35_NO_2_ by HRESIMS ([M+H]^+^, *m*/*z* 406.2751; calcd. for C_27_H_36_NO_2_, 406.2751), indicating eleven degrees of unsaturation. Comparing the ^1^H and ^13^C NMR spectral data of **9** (Figures S23 and S24) and **8** indicated that the only difference between the two structures was the disappearance of the Δ^19,20^ double bond in the C ring, which was confirmed by the ^1^H-^1^H COSY correlations of H_2_-18/H_2_-19/H_2_-20 and the HMBC correlations of H_2_-17/C-19 and H_2_-19/C-21 (Figure 2a and Figures S25–S27). The relative configuration of **9** was established by NOESY cross-peaks, which were consistent with that of **8** (Figure 2b and Figure S28). The similarity of experimental ECD curves between **8** and **9** allowed the assignment of the absolute configuration of **9** to be made as 3*S*, 4*S*, 5*S*, 8*S*, 9*S*, 16*S* (Figure 3b).

All known compounds isolated from the Δ*sclA* mutant strain were elucidated by comparing their spectral data with those reported in the literature. The compounds were identified as four usnic acid derivatives, including (−)-mycousnine (**10**), (+)-isomycousnine (**11**) [30], (−)-placodiolic acid (**12**) [31], and (±)-9-*O*-methylplacodiolic acid (**13**) [32]; five glycosylated anthraquinones, including compounds **14**–**17** [33]; two xanthones, including 4-isoprenyl ravenelin (**18**) [33] and monodictyxanthone (**19**) [34]; and three acetophenone derivatives, including 1-(2,6-dihydroxy-4-methoxy-3-methylphenyl)ethenone (**20**) [35], clavatol (**21**) [36], and clavatoic acid (**22**) [37]. The structures of these compounds are illustrated in Figure 1d. In addition to the aforementioned isolated metabolites, we performed molecular networking to investigate the low-abundance variants of different classes of metabolites, particularly cytochalasins, in the Δ*sclA* mutant. This analysis revealed the presence of many unknown metabolites that have not yet been isolated, highlighting the significant potential of this mutant for metabolite production (Figure S31).

### 2.4. Proposed Biosynthetic Pathway of Sclerin (***1***)

**Figure 4:**
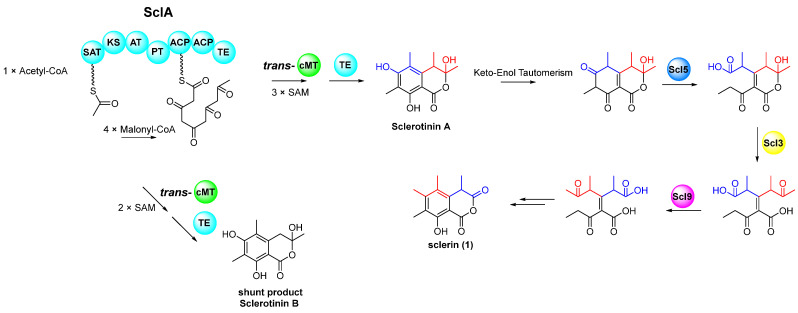
Proposed biosynthetic pathway of sclerin (**1**). SAT: starter unit acyl-carrier protein transacylase; KS: ketoacyl synthase; AT: acyltransferase; PT: product template; ACP: acyl carrier protein; TE: thioesterase; cMT: methyltransferase; SAM: *S*-adenosylmethionine.

Sclerin (**1**) is an isocoumarin phytotoxin known to cause necrosis and chlorosis in susceptible plant species and originally isolated from the pathogenic fungus *Sclerotinia sclerotiorum* [38]. To date, studies on the biosynthesis of **1** have been limited to isotope-labeling experiments [39], and no BGC responsible for its production has been reported. In this study, we inactivated the PKS gene *sclA*, which is responsible for the biosynthesis of **1**, and proposed a possible biosynthetic pathway for **1** based on gene annotations and previous reports [39,40,41]. Initially, the polyketide precursor is synthesized by SclA through the condensation of one acetyl-CoA starter unit and four malonyl-CoA extender units. During the process, the linear precursor undergoes methylation at C-4, C-5 and C-7, catalyzed by a methyltransferase using three molecules of *S*-adenosylmethionines (SAM), before being transferred to the thioesterase (TE) domain. It is worth noting that SclA comprises a starter unit acyl-carrier protein transacylase (SAT) domain, a ketoacyl synthase (KS) domain, an acyltransferase (AT) domain, a product template (PT) domain, two acyl carrier protein (ACP) domains, and a TE domain, but lacks an intrinsic methyltransferase domain. Thus, we proposed that a *trans*-methyltransferase is necessary for the methylation step, similar to the formation of pseurotin A by the *trans*-methyltransferase PsoF-MT [42]. Following methylation, the polyketide chain is released through TE-mediated condensation to produce the key intermediate sclerotinin A, along with the dimethylated byproduct sclerotinin B [43]. The subsequent cleavage of the aromatic and lactone rings of the keto-tautomer of sclerotinin A is likely catalyzed by cytochrome (CYP) P450 Scl5 and short-chain dehydrogenase (SDR) Scl3, respectively [44,45]. According to the literature [39], a mechanistically plausible isomerization of the olefinic product from the previous step is essential, which we deduced the *cis*-*trans* isomerase Scl9 is responsible for. The final anhydride product, sclerin (**1**), featuring multiple methyl substitution, is generated through a ring closure reaction (Figure 4). However, further experiments are required to validate these proposed steps.

### 2.5. Antibacterial Activities and Cytotoxicities of Compounds

The antibacterial activities of compounds **1** and **10**–**13** were tested against a panel of Gram-positive bacteria. The minimum inhibitory concentration (MIC) values are shown in Table 1. Only compounds **1** and **10** exhibited weak inhibitory activity against various Gram-positive bacteria such as *Staphylococcus aureus*, methicillin-resistant *Staphylococcus aureus* (MRSA), and *Bacillus subtilis* (Table 1).

Given the potent antitumor activity of cytochalasin-class compounds, the cell viability of all compounds was tested against five human cell lines, including human non-small-cell lung cancer cells A549, human hepatocellular liver carcinoma cell line HepG2, human glioblastoma LN229, triple-negative breast cancer MDA-MB-231, and human esophageal cancer cells TE-1, using DMSO as a control. Unfortunately, none of these compounds showed inhibitory effects on these cell lines.

## 3. Materials and Methods

### 3.1. General Experiment Procedure

NMR spectra were collected using an Avance-700 spectrometer (Bruker Company, Karlsruhe, Germany) at 700 MHz for ^1^H nuclei and 175 MHz for ^13^C nuclei. Chemical shifts (*δ*) are given with reference to tetramethylsilane (Sigma-Aldrich Inc., Vienna, Austria). Mass spectra were obtained on an Amazon SL ion trap instrument and a Maxis quadrupole-time-of-flight mass spectrometer (Bruker Company, Karlsruhe, Germany). Optical rotations were acquired using a PerkinElmer MPC 500 (PerkinElmer, Waltham, MA, USA) polarimeter. ECD spectra were measured with a Chirascan circular dichroism (Applied Photophysics Ltd., Surrey, UK). The crystallographic data were collected on an XtaLAB AFC12 (RINC): Kappa single diffractometer. UV spectra were measured with a Shimadzu UV–2550 spectrophotometer (Shimadzu, Kyoto, Japan). IR spectra were measured with an Alpha II spectrometer equipped with a platinum ATR accessory (Bruker, Karlsruhe, Germany). Medium-pressure liquid chromatography (MPLC) isolation was performed using a ComblFlash NextGen 300+ (Teledyne ISCO, Lincoln, NE, USA). Analytical HPLC was performed on an Agilent 1260 HPLC system equipped with a G7112B isocratic pump and an Agilent G7115A diode array detector (Agilent Corporation, Santa Clara, CA, USA), using a poroshell 120 EC-C18 column (2.7 µm, 3.0 × 150 mm, Agilent Corporation, Santa Clara, CA, USA). Semi-preparative HPLC was performed on an Agilent 1260 HPLC system equipped with a G1328C isocratic pump and an Agilent G7115A diode array detector (Agilent Corporation, Santa Clara, CA, USA), using an Eclipse XDB-C18 column (5 µm, 9.4 mm × 250 mm, Agilent Corporation, Santa Clara, CA, USA). Column chromatography was performed using silica gel (200–300 mesh, Qingdao Marine Chemical Corporation, Qingdao, China). All solvents used were of analytical grade (Tianjin Fuyu Chemical Co., Ltd. Tianjin, China) or chromatographic grade (Concord Technology Co., Ltd. Tianjin, China). PCR was performed on an Applied Biosystems SimpliAmpTM Thermal Cycler (Thermo Fisher Scientific, Inc., Waltham, MA, USA) with TransStart^®^ FastPfu DNA Polymerase (TransGen Biotech Co., Ltd., Beijing, China).

### 3.2. DNA/RNA Isolation, Sequencing, Manipulation, and Bioinformatic Analysis

Genomic DNA from all fungal strains was prepared using lysis buffer (50 mM Tris–HCI, pH 7.2, 50 mM EDTA, 3% SDS, 1% 2-mercaptoethanol) as previously reported [46]. Whole-genome sequencing of *M. circinata* SDU050 was performed by 2nd Illumina sequencing technologies and 3rd PacBio RS platforms in Shanghai BIOZERON biotechnology Co., LTD (Shanghai, China). The assembled genomes of *M. circinata* SDU050 cover approximately 26.5 Mb. Oligonucleotide primers used in this study (Table S5) were purchased from Qingdao Tsingke Biotechnology Co., Ltd., Qingdao, China.

The secondary metabolite BGCs of *M. circinata* SDU050 were identified and analyzed using the online software antiSMASH version 7.1.0. The sequence alignment of the gene clusters was completed by clinker software (https://cagecat.bioinformatics.nl/tools/clinker, accessed on 9 November 2023) [47].

### 3.3. HPLC Analysis

The metabolites of both strain were analyzed using a reversed-phase column poroshell 120 EC-C18 (Agilent, 2.7 µm, 3.0 × 150 mm) with a DAD detector using the solvent system (phase A, H_2_O; phase B, CH_3_CN): 0–13 min 5–100% phase B; 13–17 min 100% phase B; 17–17.01 min 100–5% phase B; 17.01–20 min 5% phase B; flow rate at 0.7 mL/min.

### 3.4. Microorganism Material, Fermentation, Extraction, and Purification

The *M. circinata* SDU050 strain was isolated from the 3448 m deep sediment of the South China Sea (Table S3). The wild-type and Δ*sclA* mutant strains of *M. circinata* SDU050 were grown on PDA plates for 7 days at 30 °C. The spores and mycelia were inoculated into 250 mL flasks containing 100 mL PDB medium and cultivated for 3 days. Subsequently, the cultures were transferred to 1 L flasks containing 250 mL of PDB medium and incubated on rotary shakers (28 °C, 200 rpm) for 8 days. The fermentation culture (6 L and 20 L for the wild-type and Δ*sclA* mutant strains, respectively) was centrifuged (3900 rpm, 15 min) to separate the supernatant and mycelium. The supernatant was extracted with ethyl acetate (8 L and 25 L for the wild-type and Δ*sclA* mutant strains, respectively) 3 times (fresh solvent for each) until no major compounds could be detected on HPLC. Residues from each extract were then combined according to HPLC-DAD analyses.

The combined extract (2.6 g) from the wild-type strain was fractionated by silica gel eluted with CH_2_Cl_2_/MeOH (1:0→0:1, *v*/*v*) to afford ten fractions (Fr. 1–10). Fr. 1–5 were combined and subjected to a silica gel CC eluted with PE/AcOEt (1:0→0:1, *v*/*v*) to obtain seven fractions (Fr. 1.1–1.7). Compounds **1** (25.9 mg, *t*_R_ = 12.1 min) and **4** (17.4 mg, *t*_R_ = 15.2 min) were obtained using HPLC (Agilent Eclipse XDB-C18 column 5 µm, 9.4 mm × 250 mm, Agilent Corporation, Santa Clara, CA, USA, MeCN/H_2_O, 64%/36% and 76%/24%, 2 mL/min, 256 nm) from Fr. 1.3 and 1.4, respectively. Fr. 7 and 8 were combined and subsequently chromatographed on a silica gel CC eluted with PE/AcOEt (1:0–0:1, *v*/*v*) to afford eight fractions (Fr. 7.1–7.8). Compounds **2** (4.4 mg, *t*_R_ = 17.0 min) and **6** (3.8 mg, *t*_R_ = 22.3 min) were further purified by semi-preparative HPLC (Agilent Eclipse XDB-C18 column 5 µm, 9.4 mm × 250 mm, Agilent Corporation, Santa Clara, CA, USA, MeCN/H_2_O with constant 0.1% formic acid, 54%/46% and 50%/50%, 2 mL/min, 267 nm and 241 nm) from Fr. 7.3 and 7.4. Fr. 7.5 and 7.6 were further purified by semi-preparative HPLC (Agilent Eclipse XDB-C18 column 5 µm, 9.4 mm × 250 mm, Agilent Corporation, Santa Clara, CA, USA, MeCN/H_2_O, 40%/60% and 54%/46%, 2 mL/min, 230 nm and 275 nm) to produce **3** (3.3 mg, *t*_R_ = 23.0 min) and **5** (11.1 mg, *t*_R_ = 17.5 min), respectively.

The combined extract (20.2 g) from the Δ*sclA* mutant strain was fractionated by silica gel eluted with CH_2_Cl_2_/MeOH (1:0→0:1, *v*/*v*). Nine fractions (Fr. 1–9) were obtained according to HPLC analysis. Fr. 1 and 2 were combined and subjected to an ODS column eluted with H_2_O/MeOH (8:2→0:1, *v*/*v*) to yield six fractions (Fr. 1.1–1.6). Fr. 1.2 and Fr. 1.3 were further fractionated by semi-preparative HPLC (Agilent Eclipse XDB-C18 column 5 µm, 9.4 mm × 250 mm, Agilent Corporation, Santa Clara, CA, USA, MeCN/H_2_O, 56%/44%, 2 mL/min, 232 nm) to produce **21** (8.4 mg, *t*_R_ = 16.8 min) and **20** (13.7 mg, *t*_R_ = 13.9 min), respectively. Fr. 1.4 was subsequently chromatographed on silica gel CC and eluted with PE/AcOEt (1:0–0:1, *v*/*v*) to obtain Fr. 1.4.1–Fr. 1.4.5. Compound **18** (9.9 mg, *t*_R_ = 18.5 min) was obtained using HPLC (Agilent Eclipse XDB-C18 column 5 µm, 9.4 mm × 250 mm, Agilent Corporation, Santa Clara, CA, USA, MeCN/H_2_O, 86%/14%, 2 mL/min, 263 nm) from Fr. 1.4.2. Compounds **9** (188.4 mg, *t*_R_ = 20.9 min) and **8** (5.1 mg, *t*_R_ = 19.7 min) were obtained using HPLC (Agilent Eclipse XDB-C18 column 5 µm, 9.4 mm × 250 mm, Agilent Corporation, Santa Clara, CA, USA, MeCN/H_2_O, 78%/22%, 2 mL/min, 218 nm) from Fr. 1.4.3. Fr. 3 was subjected to silica gel CC to afford ten subfractions (Fr. 3.1–3.10). Fr. 3.3–3.5 were combined and subsequently chromatographed on an ODS column eluted with H_2_O/MeOH (7:3→0:1, *v*/*v*) to yield six subfractions (Fr. 3.3.1–3.3.6). Compounds **7** (15.5 mg, *t*_R_ = 20.3 min), **14** (6.6 mg, *t*_R_ = 11.8 min), and **15** (7.1 mg, *t*_R_ = 16.2 min) were purified using HPLC (Agilent SB-CN Semi-Preparative column 5 µm, 9.4 mm × 250 mm, Agilent Corporation, Santa Clara, CA, USA, MeCN/H_2_O with constant 0.1% formic acid, 75%/25%, 2 mL/min, 260 nm) from Fr. 3.3.3. Fr. 3.3.4 and 3.3.5 were combined and chromatographed on HPLC (NanoChrom chromCore Phenyl column 5 µm, 10 mm × 250 mm, Nanochrom technologies Co.,Ltd., Suzhou, China, MeCN/H_2_O, 70%/30% with constant 0.1% formic acid, 2 mL/min, 432 nm) to obtain **16** (7.4 mg, *t*_R_ = 18.5 min) and **17** (2.3 mg, *t*_R_ = 13.1 min). Fr. 3.6–3.9 were combined and subsequently subjected to an ODS column eluted with H_2_O/MeOH (8:2→0:1, *v*/*v*) to afford eight fractions (Fr. 3.6.1–3.6.8). Compounds **19** (3.2 mg, *t*_R_ = 20.5 min) and **22** (99.1 mg, *t*_R_ = 10.6 min) were yielded by HPLC (Agilent Eclipse XDB-C18 column 5 µm, 9.4 mm × 250 mm, Agilent Corporation, Santa Clara, CA, USA, MeCN/H_2_O, 55%/45%, 2 mL/min, 254 nm) from Fr. 3.6.2. Compounds **10** (17.8 mg, *t*_R_ = 20.1 min), **11** (7.5 mg, *t*_R_ = 19.4 min), **12** (10.9 mg, *t*_R_ = 17.8 min) and **13** (8.8 mg, *t*_R_ = 22.5 min) were purified using HPLC (Agilent SB-CN Semi-Preparative column 5 µm, 9.4 mm × 250 mm, Agilent Corporation, Santa Clara, CA, USA, MeCN/H_2_O, 68%/32%, 2 mL/min, 280 nm) from Fr. 3.6.4–3.6.7.

### 3.5. Characterization of Compounds

Malcirchalasin A (**7**): colorless solid; m.p. 74–76 °C; ${\left[\alpha \right]}_{D}^{20}$ +134.26 (*c* 0.10, MeOH); UV (MeOH) *λ*_max_ (log ε) 217 (2.65) nm; IR: 3226, 2923, 2874, 1700, 1454, 1378, 1115, 982, 700 cm^−1^; ^1^H and ^13^C NMR spectral data, see Table S1; HRESIMS [M+H]^+^, *m*/*z* 420.2539; (calcd. for C_27_H_34_NO_3_, 420.2533); ECD (*c* 0.025, MeOH) *λ*_max_ (Δ*ε*) 238 (+10.36), 289 (−4.41) nm.

Malcirchalasin B (**8**): white solid; m.p. 130–132 °C; ${\left[\alpha \right]}_{D}^{20}$ +10.02 (*c* 0.10, MeOH); UV (MeOH) *λ*_max_ (log ε) 218 (2.83) nm; IR: 3270, 2925, 2855, 1693, 1621, 1454, 1378, 1090, 977, 700 cm^−1^; ^1^H and ^13^C NMR spectral data, see Table S1; HRESIMS [M+H]^+^, *m*/*z* 404.2590; (calcd. for C_27_H_34_NO_2_, 404.2584); ECD (*c* 0.025, MeOH) *λ*_max_ (Δ*ε*) 224 (−2.65) nm, 242 (+3.85).

Malcirchalasin C (**9**): white paste; ${\left[\alpha \right]}_{D}^{20}$ +30.01 (*c* 0.10, MeOH); UV (MeOH) *λ*_max_ (log ε) 224 (3.12) nm; IR: 3234, 2917, 2874, 1685, 1454, 1380, 1302, 1049, 979, 700, 612 cm^−1^; ^1^H and ^13^C NMR spectral data, see Table S1; HRESIMS [M+H]^+^, *m*/*z* 406.2751; (calcd. for C_27_H_36_NO_2_, 406.2751); ECD (*c* 0.025, MeOH) *λ*_max_ (Δ*ε*) 232 (+5.79).

### 3.6. Antibacterial Activity Assays

The antibacterial activity of compounds **1** and **10**–**13** was tested following the standard protocol provided by the Clinical and Laboratory Standards Institute (CLSI) [48]. Eight bacterial strains were used, including *Staphylococcus aureus* ATCC 29213, *Staphylococcus aureus* 16339, MRSA, *Enterococcus faecalis* ATCC 29212, *Bacillus subtilis* BS01, *Staphylococcus simulans* AKA1, *Enterococcus gallinarum* 5F52C, and *Staphylococcus epidermidis* SRH–sep. Compounds **1** and **10**–**13** were dissolved in dimethyl sulfoxide (DMSO) at a concentration of 3.2 mg/mL. Vancomycin and ampicillin were used as antibacterial control agents. After incubation at 37 °C for 16 h, a microdilution instrument was utilized to determine the minimum concentration of each tested compound that completely inhibited bacterial growth in the microdilution wells. All assays were performed in triplicate.

### 3.7. Cytotoxic Activity Assays

All tumor cells were plated on a 96-well plate at densities of 3000, 3000, 3000, and 5000 cells per well, respectively, and cultured at 37 °C and 5% CO_2_ with saturated humidity overnight. The indicated compounds were added to each cell line in triplicate at a final concentration of 10 μM. Doxorubicin (10 μM) was used as a positive control and DMSO was used as a negative control. After a 72 h of treatment, 10 μL of CCK-8 reagent was added directly to each well and incubated for an additional 4 h. Absorbance was measured at 450 nm with a reference wavelength of 600 nm. The relative cell viability was calculated as follows:

Relative cell viability (%) = (Sample absorbance − Culture medium absorbance)/(Negative control absorbance − Culture medium absorbance) × 100.

### 3.8. Preparation of M. circinata SDU050 Protoplasts

The general fungal transformation method has been previously described in detail elsewhere [49]. For the transformation of *M. circinata* SDU050, spores were cultured in 50 mL PDB broth in a 250 mL flask at 28 °C with shaking at 200 rpm for approximately 12 h. Germination was confirmed by microscopic examination, after which the spores were harvested and rinsed with 20 mL of osmotic buffer (Table S6). Lysing enzyme (2 mg/mL, Sigma Aldrich, Darmstadt, Germany) and Yatalase (3 mg/mL, Takara, Osaka, Japan) were used to prepare the protoplasts. Protoplasts were prepared by incubating the mixture in 10 mL osmotic buffer at 30 °C, 80 rpm for 8 h. The mixture was collected and transformed into a 30 mL sterile glass tube and overlaid carefully with 10 mL trapping buffer. After centrifugation (3900 rpm, 40 min, 4 °C), the protoplasts at the interface were carefully transformed into a 50 mL sterile tube and washed with 10 mL STC buffer.

### 3.9. Construction of CRISPR-Cas9 Plasmids and Fungal Transformation

The construction of CRISPR-Cas9 plasmids followed a previously described method [50]. To construct plasmids (Table S4) for the Cas9-based gene disruption system, two overlapped fragments for the overexpression of the single guide RNA (sgRNA) containing the target gene-specific protospacer sequence and the sgRNA scaffold sequence were amplified by PCR with two sets of primers of Cas9-sgDNA F/R and Cas9-*sclA* F1/R1 using pFC332 as the template [51]. The fragments were inserted into the BsaI-digested pBSKII-Cas9-hph vector by a Seamless Cloning and Assembly Kit (TransGen, Beijing, China) and were introduced into commercial *E. coli* DH5*α* by transformation. The plasmids were then sequenced to confirm identities and named pCas9-*sclA*. The gene-specific protospacer sequence was designed using an online website (http://www.e-crisp.org/, accessed on 22 December 2023).

The transformation of *M. circinata* SDU050 with the constructed gene inactivation plasmids was performed by the previously reported protoplast–polyethylene glycol method, followed by CRISPR-Cas9-guided homologous recombination [52]. The transformants created in this study are provided in Table S3.

### 3.10. ECD Calculations

Conformational searches were performed with the Spartan’14 software (Wavefunction Inc., Irvine, CA, USA) using the Merck Molecular Force Field (MMFF) method as previously described [53]. The MMFF minima were then reoptimized with density functional theory (DFT) calculations at the B3LYP/6–31 + g (d, p) level using the polarizable continuum model (PCM) with Gaussian 16 software. The theoretical ECD calculations were further conducted in MeOH by time-dependent DFT (TDDFT) at the B3LYP/6–31 + g (d, p) level for the low-energy conformers using 50 excited states. ECD spectra were generated using the program SpecDis 1.7 (University of Würzburg) and Prism 5.0 (Graph Pad Software Inc., San Diego, CA, USA) with a half-bandwidth of 0.3 eV, according to the Boltzmann-calculated contribution of each conformer after UV correction.

## 4. Conclusions

In summary, deep-sea fungi hold significant potential for producing structurally diverse and biologically active secondary metabolites. To activate or enhance the production of previously undescribed metabolites, various strategies have been developed. In the exploration of the chemical diversity of the deep-sea sediment-derived fungus *M. circinata* SDU050, six compounds were isolated and identified from PDB shaking cultivation. The whole-genome sequencing of *M. circinata* SDU050 revealed at least 69 biosynthetic gene clusters, including 30 T1PKSs, highlighting its potential to produce a wide range of secondary metabolites. To uncover cryptic or lowly expressed metabolites not detected under standard culture conditions, we employed a metabolic blockade-based genome mining strategy by disrupting the biosynthesis of sclerin (**1**), which successfully led to the production of three new cytochalasins with **9** as the major product (9.5 mg/L) and **7** and **8** as minor metabolites (<1.0 mg/L). Thirteen known compounds exhibited diverse structures and biological activities. Antibacterial assays showed that compounds **1** and **10** exhibited weak inhibitory activity against several Gram-positive bacteria. Furthermore, we proposed the biosynthetic pathway for sclerin (**1**), which lays the groundwork for future studies on the biosynthetic mechanisms of this phytotoxin. Our work offers valuable insights not only for addressing unresolved biosynthetic questions for these compounds, but also for exploring cryptic secondary metabolites with biological activities from deep-sea fungi.

## Figures and Tables

**Table 1:** Antibacterial activity of compounds **1**, **10**–**13** against bacterial strains (MIC, µg/mL).

Bacterial Strains	MIC (µg/mL) of Standard		Diameters (mm) of Compounds 1, 10–13				
	Vancomycin	Ampicillin	1	10	11	12	13
*Staphylococcus aureus* ATCC 29213	0.5	1	64	64	>64	>64	>64
*Staphylococcus aureus* 16339	1	64	>64	32	>64	>64	>64
MRSA	1	64	>64	64	>64	>64	>64
*Enterococcus faecalis* ATCC 29212	1	1	>64	>64	>64	>64	>64
*Bacillus subtilis* BS01	0.25	0.25	64	32	>64	>64	>64
*Staphylococcus simulans* AKA1	1	>64	>64	>64	>64	>64	>64
*Enterococcus gallinarum* 5F52C	4	8	>64	>64	>64	>64	>64
*Staphylococcus epidermidis* SRH–sep	8	64	>64	>64	>64	>64	>64

Vancomycin and ampicillin were used as positive controls.

## Data Availability

The authors declare that all relevant data supporting the findings of this study are available within the article and its Supplementary Materials files or from the corresponding authors upon request.

## Supplementary Materials

The following supporting information can be downloaded at https://www.mdpi.com/article/10.3390/md23010050/s1, Figure S1: ORTEP drawing of compound **7** with 50% ellipsoid contour probability; Figure S2: Construction and gel electrophoresis analyses of mutant Δ*sclA*; Figure S3: The organization of the PKS/NRPS BGC responsible for the biosynthesis of cytochalasins validated by the gene inactivation; Figures S4–S28: HRESIMS, IR, and NMR data of compounds **7**–**9**; Figure S29: The optimized conformers and equilibrium populations of **7**; Figure S30: The optimized conformers and equilibrium populations of **8**; Figure S31: Molecular network of secondary metabolites in extracts of *Malbranchea circinata* SDU050; Table S1: The antiSMASH-predicted BGCs for *M. circinata* SDU050; Table S2: Deduced functions of *orfs* in the *scl* BGC; Table S3: Strains used and constructed in this study; Table S4: Plasmids used in this study; Table S5: Primers used in this study; Table S6: Media and stock used in this study; Table S7: Summary of ^1^H and ^13^C NMR data for compounds **7**–**9** (*δ* in ppm); Table S8: Single-crystal X-ray diffraction analysis data of compound **7**.
