# Supplementary material for: Metabolic Blockade-Based Genome Mining of Malbranchea circinata SDU050: Discovery of Diverse Secondary Metabolites
Source: Mar Drugs. 2025 Jan 20;23(1):50. doi: 10.3390/md23010050 (PMC11766578; doi:10.3390/md23010050)

Supplementary Materials

Table of Contents

Table S1. The antiSMASH-predicted BGCs for *Malbranchea circinata* SDU050. 1
Table S2. Deduced functions of *orfs* in the *scl* BGC. 4
Table S3. Strains used and constructed in this study. 5
Table S4. Plasmids used in this study. 5
Table S5. Primers used in this study. 5
Table S6. Media and stock used in this study. 6
Table S7: Summary of ^{1}H and ^{13}C NMR data for compounds **7**-**9** in CD_3OD and $CDCl_3$ (δ in ppm) 7
Table S8. Single crystal X-ray diffraction analysis data of compound **7**. 8
Figure S1. ORTEP drawing of compound **7** with 50% ellipsoid contour probability. 9
Figure S2. Construction and gel electrophoresis analyses of mutant Δ*sclA* 10
Figure S3. The organization of the PKS/NRPS BGC responsible for the biosynthesis of cytochalasins validated by the gene inactivation. 10
Figure S4. (+)-HRESIMS spectrum of Malcirchalasin A (**7**). 11
Figure S5. IR spectrum of Malcirchalasin A (**7**). 11
Figure S6. ^{1}H NMR (700 MHz, chloroform-*d*) spectrum of Malcirchalasin A (**7**). 12
Figure S7. ^{13}C NMR (175 MHz, chloroform-*d*) spectrum of Malcirchalasin A (**7**). 12
Figure S8. DEPT 135 (175 MHz, chloroform-*d*) spectrum of Malcirchalasin A (**7**). 13
Figure S9. ^{1}H-^{1}H COSY spectrum of Malcirchalasin A (**7**). 13
Figure S10. HSQC spectrum of Malcirchalasin A (**7**). 14
Figure S11. HMBC spectrum of Malcirchalasin A (**7**). 14
Figure S12. NOESY spectrum of Malcirchalasin A (**7**). 15
Figure S13. (+)-HRESIMS spectrum of Malcirchalasin B (**8**). 16
Figure S14. IR spectrum of Malcirchalasin B (**8**) 16
Figure S15. ^{1}H NMR (700 MHz, methanol-d_4) spectrum of Malcirchalasin B (**8**). 17
Figure S16. ^{13}C NMR (175 MHz, methanol-d_4) spectrum of Malcirchalasin B (**8**). 17
Figure S17. ^{1}H-^{1}H COSY spectrum of Malcirchalasin B (**8**). 18
Figure S18. HSQC spectrum of Malcirchalasin B (**8**). 18
Figure S19. HMBC spectrum of Malcirchalasin B (**8**). 19
Figure S20. NOESY spectrum of Malcirchalasin B (**8**). 19
Figure S21. (+)-HRESIMS spectrum of Malcirchalasin C (**9**) 20
Figure S22. IR spectrum of Malcirchalasin C (**9**). 20
Figure S23. ^{1}H NMR (700 MHz, methanol-d_4) spectrum of Malcirchalasin C (**9**). 21
Figure S24. ^{13}C NMR (175 MHz, methanol-d_4) spectrum of Malcirchalasin C (**9**). 21
Figure S25. ^{1}H-^{1}H COSY spectrum of Malcirchalasin C (**9**). 22
Figure S26. HSQC spectrum of Malcirchalasin C (**9**). 22
Figure S27. HMBC spectrum of Malcirchalasin C (**9**). 23
Figure S28. NOESY spectrum of Malcirchalasin C (**9**) 23
Figure S29. The optimized conformers and equilibrium populations of **7** 24
Figure S30. The optimized conformers and equilibrium populations of **8** 25
Figure S31. Molecular network of secondary metabolites in extracts of *Malbranchea circinata* SDU050. 26

Table S1. The antiSMASH-predicted BGCs for *Malbranchea circinata* SDU050.

BGC	Region	Type	Position		Product(SI>50%)
			From	To	
Cluster 1	1.1	NRPS, T1PKS	1	50,535	
Cluster 2	1.2	T1PKS	121,431	168,808	
Cluster 3	1.3	T1PKS	794,692	844,560	
Cluster 4	1.4	NRPS-like	1,022,514	1,065,976	
Cluster 5	1.5	T1PKS	1,397,638	1,450,769	
Cluster 6	1.5	fungal-RiPP-like	1,450,770	1,511,746	
Cluster 7	1.6	T1PKS	1,773,941	1,821,995	trichoxide
Cluster 8	1.7	NRPS, T1PKS	1,945,159	2,001,432	
Cluster 9	1.8	indole	2,198,180	2,219,523	
Cluster 10	1.9	T1PKS	2,573,081	2,620,957	AKML A/B/C/D
Cluster 11	1.10	T1PKS	2,763,863	2,810,887	
Cluster 12	1.11	terpene	2,909,815	2,931,766	
Cluster 13	1.12	NRPS	3,193,707	3,248,244	
Cluster 14	1.13	fungal-RiPP-like	3,340,401	3,402,658	
Cluster 15	1.14	T1PKS	4,878,820	4,926,029	
Cluster 16	1.15	T1PKS	4,964,161	5,010,815	
Cluster 17	1.16	T1PKS	5,063,751	5,110,489	
Cluster 18	1.17	T1PKS	5,660,052	5,708,440	
Cluster 19	1.17	NRPS-like	5,685,708	5,728,140	
Cluster 20	1.18	T1PKS	5,792,601	5,844,816	
Cluster 21	2.1	T1PKS	129,484	175,036	
Cluster 22	2.2	terpene	892,434	914,449	
Cluster 23	2.3	fungal-RiPP-like	1,173,957	1,238,502	
Cluster 24	2.4	T1PKS	1,630,963	1,678,916	
Cluster 25	2.4	T1PKS	1,677,462	1,729,479	
Cluster 26	2.5	NRPS-like	2,287,944	2,331,353	

Cluster 27	2.6	fungal-RiPP-like	2,701,543	2,762,459	
Cluster 28	2.7	terpene	2,893,577	2,914,761	
Cluster 29	2.8	fungal-RiPP-like	3,306,992	3,388,685	
Cluster 30	2.8	NRPS	3,343,343	3,408,685	
Cluster 31	3.1	NRPS-like, T1PKS	192,668	240,191	swainsonine
Cluster 32	3.2	T1PKS	1,208,456	1,257,041	
Cluster 33	3.3	T1PKS	3,555,328	3,616,474	
Cluster 34	4.1	T1PKS	13,556	61,375	
Cluster 35	4.1	T1PKS	57,452	127,824	
Cluster 36	4.2	indole	401,825	423,255	
Cluster 37	4.3	NRPS-like	743,573	789,156	
Cluster 38	4.3	NRPS-like	752,259	796,183	
Cluster 39	4.4	fungal-RiPP-like	1,073,005	1,135,667	
Cluster 40	4.5	T1PKS	1,175,205	1,224,695	
Cluster 41	4.6	T1PKS	1,487,335	1,536,244	
Cluster 42	4.7	terpene	1,799,121	1,821,407	clavaric acid
Cluster 43	5.1	fungal-RiPP-like	1	39,765	
Cluster 44	5.1	fungal-RiPP-like	1,545	62,033	
Cluster 45	5.2	fungal-RiPP-like	251,540	313,918	
Cluster 46	5.3	NRPS-like	1,468,983	1,517,847	
Cluster 47	5.4	NRPS	2,395,753	2,459,739	
Cluster 48	5.5	T1PKS	2,524,143	2,572,480	
Cluster 49	5.6	NRPS-like	4,066,662	4,110,077	
Cluster 50	5.6	T1PKS	4,078,883	4,126,784	
Cluster 51	5.7	T1PKS	4,353,201	4,400,379	
Cluster 52	5.8	fungal-RiPP-like	4,867,925	4,928,903	
Cluster 53	5.9	T1PKS	4,941,967	4,988,605	fujikurin A/B/C/D
Cluster 54	6.1	T1PKS	197,820	246,822	
Cluster 55	6.2	NRPS-like	477,497	521,107	

Cluster 56	6.3	fungal-RiPP-like	579,264	640,216	
Cluster 57	6.4	T1PKS	689,938	737,480	
Cluster58	6.5	NRPS	1,456,143	1,510,753	
Cluster 59	6.6	T1PKS	1,775,183	1,823,550	cichorine
Cluster 60	6.7	T1PKS	2,370,832	2,419,010	
Cluster 61	7.1	terpene	48,162	68,962	
Cluster 62	8.1	indole	388,727	410,602	
Cluster 63	8.1	T1PKS	407,085	454,116	
Cluster 64	8.1	NRPS-like	443,558	486,402	
Cluster 65	8.2	NRPS, T1PKS	597,132	648,233	
Cluster 66	8.3	T1PKS	898,191	956,862	Sorbicillin/ bisvertinolone
Cluster 67	8.3	NRPS	932,687	989,242	
Cluster 68	9.1	fungal-RiPP-like	468,915	528,215	
Cluster 69	9.1	NRPS-like	487,893	532,312	

Table S2. Deduced functions of *orfs* in the *scl* BGC (accession number: PQ824967).

orfs	Size (AA)	Proposed function	ID/SI	Origin and protein homologue
1	1147	transcription factor Cmr1	44/74	*Bombardia bombarda*/KAK0615866.1
2	275	versicolorin reductase	77/99	*Lachnellula suecica*/TVY85119.1
3	265	transcription factor	47/26	*Diplocarpon rosae*/PBP24182.1
A	2142	BcPKS12, polyketide synthase	65/97	*Calycina marina*/KAG9246027.1
5	543	cytochrome P450 monooxygenase psoD	74/89	*Hyphodiscus hymeniophilus*/KAG0648686.1
6	246	4-coumarate-coA ligase 1	75/99	*Physcia stellaris*/KAG7007251.1
7	245	acetyl-CoA synthetase-like protein	82/100	*Stipitochalara longipes*/KAE9381253.1
8	854	fungal-specific transcription factor	60/100	*Hyaloscypha finlandica*/KAH8770291.1
9	600	peptidyl-prolyl cis-trans isomerase cyp15	89/92	*Lachnellula cervine*/TVY53092.1
10	410	cyclin-domain-containing protein	60/99	*Bisporella* sp./KAH8602979.1
11	275	nucleophile aminohydrolase	57/69	*Calycina marina*/KAG9245827.1
12	204	FAD dependent sulfhydryl oxidase Erv2	72/88	*Halenospora varia*/KAH6671069.1

Table S3. Strains used and constructed in this study.

Strains	Description	Reference or source
Bacteria Strains		
E. coli DH5α	Host strain of general cloning	Sangon Biotech
Fungal Strains		
M. circinata SDU050	Wild strain	This study
M. circinata SDU050/Δ*sclA*	*sclA* disruption mutant strain originated from *M. circinata* SDU050	This study

Table S4. Plasmids used in this study.

Plasmid	Vector	Genes/Description
pBSKII-cas9-hph	-	The plasmid can be BsaI digested as CRISPR-Cas9 plasmid vector
pFC332	-	Template for sgDNA
pCas9-*sclA*	pBSKII-cas9-hph	P_{gpdA}-*HH-repeat-protospacer-sgDNA-HDV*-T_{trpC}

Table S5. Primers used in this study.

Primer	Sequence (5'-3')
**For plasmids construction**	
pCas9-ligase-F	TCAGGGCGATGGCCCACTACGCGTAAGCTCCCTAATTGGCC
pCas9-ligase-R	TGATTAGGGTGATGGTTCACGAGCCAAGAGCGGATTCCTCAG
pCas9-*sclA*-F	ACGAGTAAGCTCGTCGATCTGTCTGATAAGCCTCTGTTTTAGAGCTAGAAATAGCAAG
pCas9-*sclA*-R	GACGAGCTTACTCGTTTCGTCCTCACGGACTCATCAGGATCTGCGGTGATGTCTGCTC
**For confirmation**	
ID-Cas9-ligase-F	TCAGGGCGATGGCCCACTACGCGTAAGCTCCCTAATTGGCC
ID-Cas9-ligase-R	TGATTAGGGTGATGGTTCACGAGCCAAGAGCGGATTCCTCAG
ID-*sclA*-up-F1	GATCTGTCTGATAAGCCTCT
ID-*sclA*-up-F2	GCGCATGGGCCTTTCTTTGATAG
ID- *sclA*-down-R	GCCCAAGGGTATGGATCATCGAA

Table S6. Media and stock used in this study.

Medium name	Ingredient
Potato Dextrose Broth (PDB) Medium (1 L)<br>(made by HUANKAI MICROBIAL SCL&TECH.CO., LTD, China)	24 g PDB power
Potato Dextrose Agar (PDA) Medium (1 L)<br>(made by HUANKAI MICROBIAL SCL&TECH.CO., LTD, China)	39 g PDA power
LB Medium (1 L)	5 g Yeast extract<br>10 g Tryptone<br>10 g NaCl<br>Adjust pH to 7.2–7.4 with 1M NaOH
Protoplast Medium (1 L)	1 M sucrose<br>10 g Dextrose/Glucose<br>20 mL Salt solution
Lysis Buffer	50 mM EDTA, 3% SDS, 50 mM Tris-HCl, pH<br>7.2, 1% 2-mercaptoethanol
Osmotic Buffer	1.2 M $MgSO_4 \cdot 7H_2O$, 10 mM NaPhosphate buffer
Trapping Buffer	0.6 M sorbitol, 0.1 M Tris HCl, pH 7.0
STC Buffer	1.2 M sorbitol, 10 mM $CaCl_2$, 10 mM Tris-HCl pH 7.5
PEG solution	60% PEG, 50 mM $CaCl_2$, and 50 mM Tris-HCl, pH 7.5
Salt solution (1L)	26 g KCl<br>76 g KH_2PO_4<br>26 g $MgSO_4$<br>50 mL Trace element<br>Add 2 mL chloroform per L of solution
Trace elements (100 mL)	2.20 g $ZnSO_4 \bullet 7H_2O$<br>1.10 g H_3BO_3<br>0.50 g $MnCl_2 \bullet 4H_2O$<br>0.16 g $FeSO_4 \bullet 7H_2O$<br>0.16 g $CoCl_2 \bullet 5H_2O$<br>0.16 g $CuSO_4 \bullet 5H_2O$<br>0.11 g $(NH_4)_6Mo_7O_{24} \bullet 4H_2O$<br>Adjusted pH to 6.5 with 1 M KOH

Table S7: Summary of ^{1}H and ^{13}C NMR data for compounds **7-9** in CD_3OD and $CDCl_3$ (δ in ppm).

no.	**7** ($CDCl_3$)			**8** (CD_3OD)			**9** (CD_3OD)		
	δ_H (mult., *J*)	type	δ_C	δ_H (mult., *J*)	type	δ_C	δ_H (mult., *J*)	type	δ_C
1		C	174.3		C	175.9		C	177.4
2		NH			NH			NH	
3	3.51, m	CH	58.1	3.33, m (overlap)	CH	55.8	3.46, m	CH	54.9
4	3.56, m (overlap)	CH	49.8	3.12, dd (5.2, 3.5)	CH	49.1	2.75, dd (5.8, 2.5)	CH	50.7
5		C	147.7	2.30, m	CH	36.1	2.43, m	CH	36.4
6		C	133.4		C	142.2		C	140.7
7		C	195.8	5.38, m	CH	126.5	5.37, m	CH	126.7
8	3.26, dd (10.0, 2.6)	CH	54.5	2.43, d (9.9)	CH	50.2	2.75, m (overlap)	CH	51.0
9		C	61.6		C	69.6		C	68.5
10	2.76, dd (13.3, 6.6)	CH_2	42.2	2.72, dd (13.5, 3.7)	CH_2	44.5	2.71, dd (13.5, 3.7)	CH_2	43.5
	2.50, dd (13.3, 8.6)			2.63, dd (13.5, 9.0)			2.61, dd (13.5, 9.0)		
11	1.56, s	CH_3	19.0	1.06, d (7.3)	CH_3	14.0	1.11, d (7.3)	CH_3	13.6
12	1.70, s	CH_3	12.1	1.77, s	CH_3	20.1	1.75, s	CH_3	20.0
13	6.36, dd (15.6, 9.9)	CH	124.2	5.76, dd (15.5, 10.0)	CH	132.4	6.18, dd (15.2, 10.3)	CH	130.5
14	5.50, ddd (15.0, 10.4, 4.1)	CH	134.6	5.29, ddd (15.5, 10.0, 5.6)	CH	135.8	5.44, ddd (15.2, 10.3, 4.7)	CH	131.8
15	2.37, m	CH_2	40.7	2.02, m	CH_2	41.7	2.17, m	CH_2	41.5
	2.01, m			1.67, m			1.88, m		
16	2.00, m	CH	30.4	1.59, m	CH	39.0	1.93, m	CH	32.1
17	1.44, m	CH_2	32.2	1.53, m	CH_2	35.6	1.35, m	CH_2	33.1
	1.32, m			1.42, m			1.25, m		
18	1.36, m	CH_2	21.8	2.33, m	CH_2	33.8	1.17, m	CH_2	23.0
	1.21, m			1.87, m			1.13, m		
19	2.05, m	CH_2	22.3	6.29, ddd (16.2, 10.8, 5.8)	CH	147.3	1.66, m	CH_2	22.9
20	3.56, m (overlap)	CH_2	39.7	6.82, d (16.2)	CH	132.8	3.24, m	CH_2	39.6
	2.28, ddd (18.5, 6.1, 2.7)						1.13, m		
21		C	209.0			201.1		C	213.5
22	0.90, d (6.3)	CH_3	18.7	1.02, d (7.2)	CH_3	25.2	0.87, d (7.2)	CH_3	19.2
1′		C	136.5		C	138.0		C	137.3
2′, 6′	7.16, d (7.3)	CH	129.3	7.14, d (7.2)	CH	131.2	7.10, d (7.2)	CH	131.9
3′, 5′	7.35, t (7.3)	CH	129.1	7.25, t (7.2)	CH	129.5	7.24, t (7.2)	CH	129.4
4′	7.28, t (7.3)	CH	127.4	7.17, t (7.2)	CH	127.7	7.18, t (7.2)	CH	127.6

Recorded at 700 (for ^{1}H) or 175 (for ^{13}C) MHz. Coupling constant *J* is in Hz and overlapped signals are reported without designating multiplicity.

Table S8. Single crystal X-ray diffraction analysis data of compound **7**.

**Identification code**	**7**
Empirical formula	$C_{27}H_{33}NO_3$
Formula weight	419.54
Temperature/K	100(1)
Crystal system	trigonal
Space group	P3221
a/Å	12.2833(2)
b/Å	12.2833(2)
c/Å	27.4964(5)
α/°	90
β/°	90
γ/°	120
Volume/Å3	3592.83(13)
Z	6
ρcalcg/cm3	1.163
μ/mm-1	0.590
F(000)	1356.0
Crystal size/mm3	0.2 × 0.15 × 0.13
Radiation	Cu Kα (λ = 1.54184)
2Θ range for data collection/°	8.312 to 147.5
Index ranges	-14 ≤ h ≤ 14, -11 ≤ k ≤ 15, -32 ≤ l ≤ 33
Reflections collected	12496
Independent reflections	4724 [Rint = 0.0528, Rsigma = 0.0496]
Data/restraints/parameters	4724/267/347
Goodness-of-fit on F2	1.066
Final R indexes [I>=2σ (I)]	R1 = 0.0645, wR2 = 0.1637
Final R indexes [all data]	R1 = 0.0703, wR2 = 0.1670
Largest diff. peak/hole / e Å-3	0.20/-0.19
Flack parameter	0.3(3)

Figure S1. ORTEP drawing of compound **7** with 50% ellipsoid contour probability.

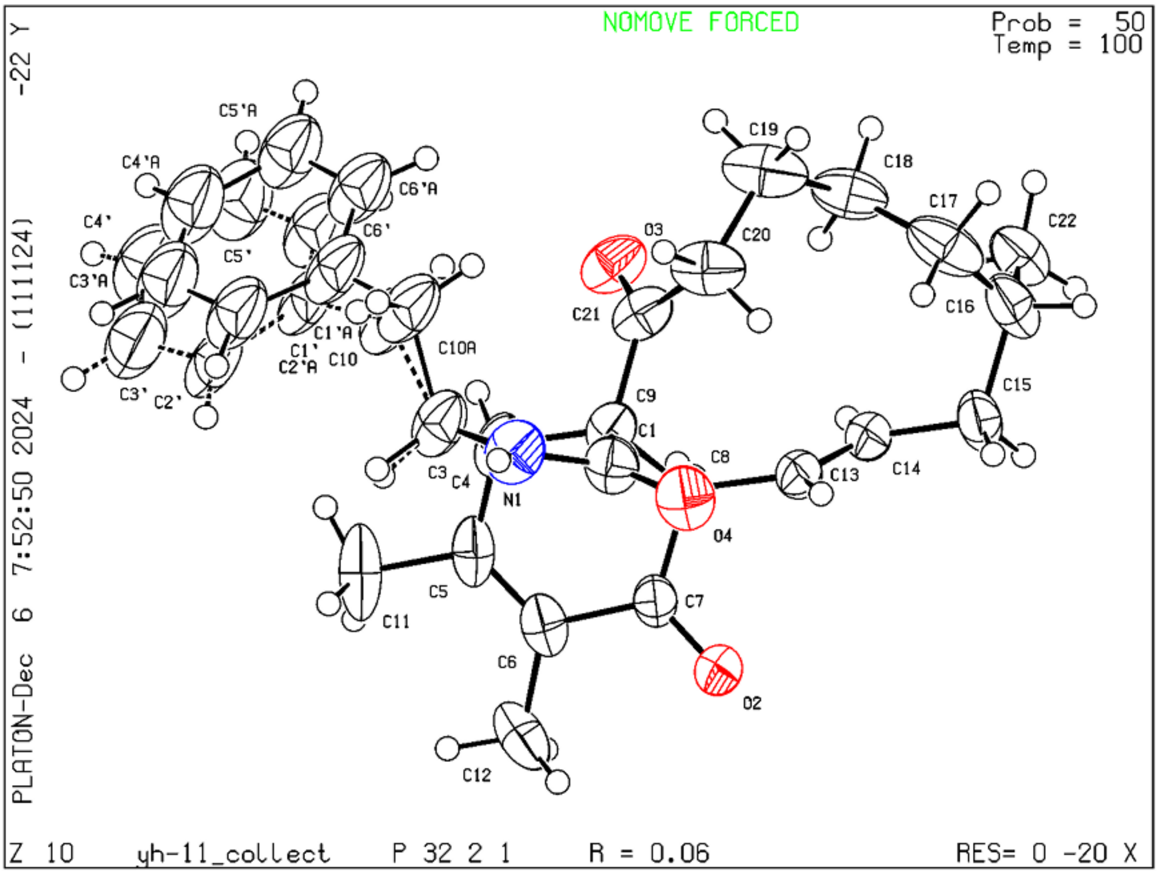

Figure S2. Construction and gel electrophoresis analyses of mutant Δ*sclA*. (a) Construction of Δ*sclA* mutant and predicted PCR fragment size from wild-type and mutant. (b) Verification of the Δ*sclA* mutant by PCR DNA templates from WT (line 1 and 3) and *ΔsclA* mutant (line 2 and 4). Marker, DNA marker DL5000 (lane M). The long plasmid fragment inserted at the target site is difficult to be amplified**.**

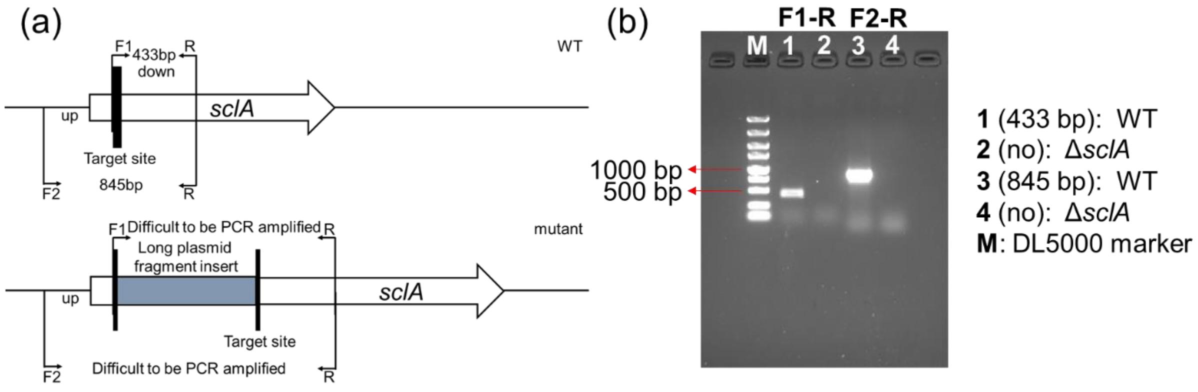

Figure S3. The organization of the PKS/NRPS BGC responsible for the biosynthesis of cytochalasins validated by the gene inactivation.

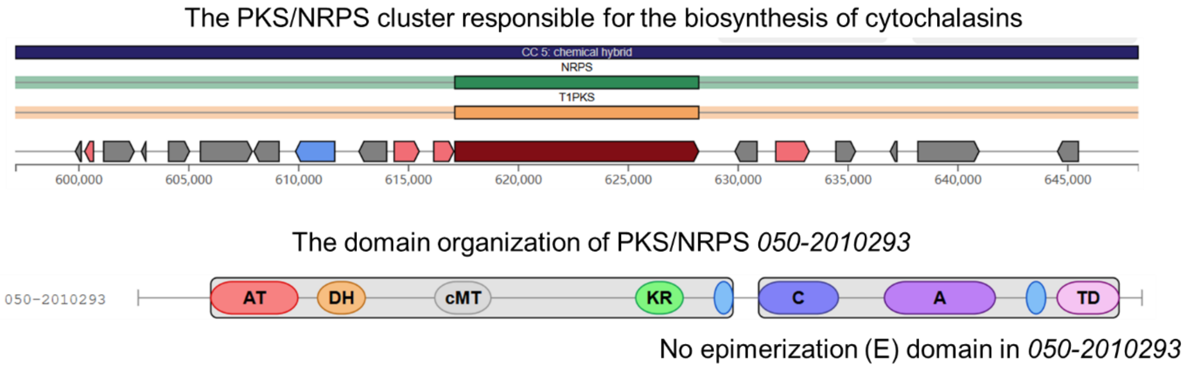

Figure S4. (+)-HRESIMS spectrum of Malcirchalasin A (**7**).

Mass Spectrum SmartFormula Report

Analysis Info

Analysis Name	D:\Data\MS\data\202409\yanghu_3036-11_pos_31_01_18235.d	Acquisition Date	9/26/2024 5:27:39 PM
Method	LC_Direct Infusion_pos_70-500mz.m	Operator	SCSIO
Sample Name	yanghu_3036-11_pos	Instrument	maXis 255552.00029
Comment			

Acquisition Parameter

Source Type	ESI	Ion Polarity	Positive	Set Nebulizer	0.4 Bar
Focus	Active	Set Capillary	4500 V	Set Dry Heater	180 °C
Scan Begin	70 m/z	Set End Plate Offset	-500 V	Set Dry Gas	4.0 l/min
Scan End	1500 m/z	Set Charging Voltage	0 V	Set Divert Valve	Waste
		Set Corona	0 nA	Set APCI Heater	0 °C

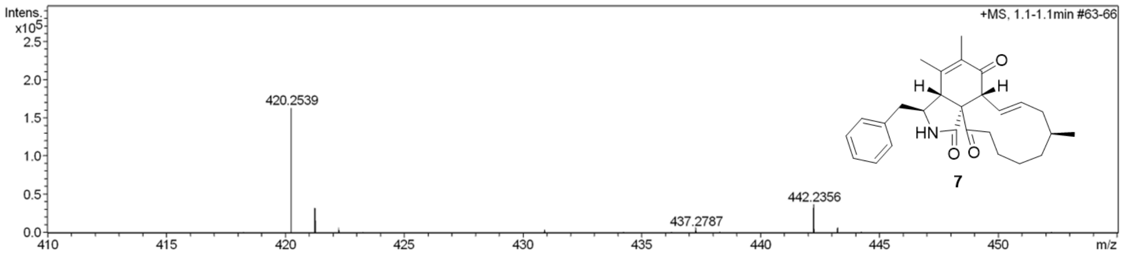

Meas. m/z	#	Ion Formula	Score	m/z	err [ppm]	err [mDa]	mSigma	rdb	e⁻ Conf	N-Rule
420.2539	1	C27H34NO3	100.00	420.2533	1.3	0.6	53.0	11.5	even	ok
442.2356	1	C27H33NNaO3	100.00	442.2353	0.8	0.4	62.7	11.5	even	ok
839.4997	1	C54H67N2O6	100.00	839.4994	0.4	0.3	41.4	22.5	even	ok
861.4811	1	C54H66N2NaO6	100.00	861.4813	0.3	0.2	66.9	22.5	even	ok

Figure S5. IR spectrum of Malcirchalasin A (**7**).

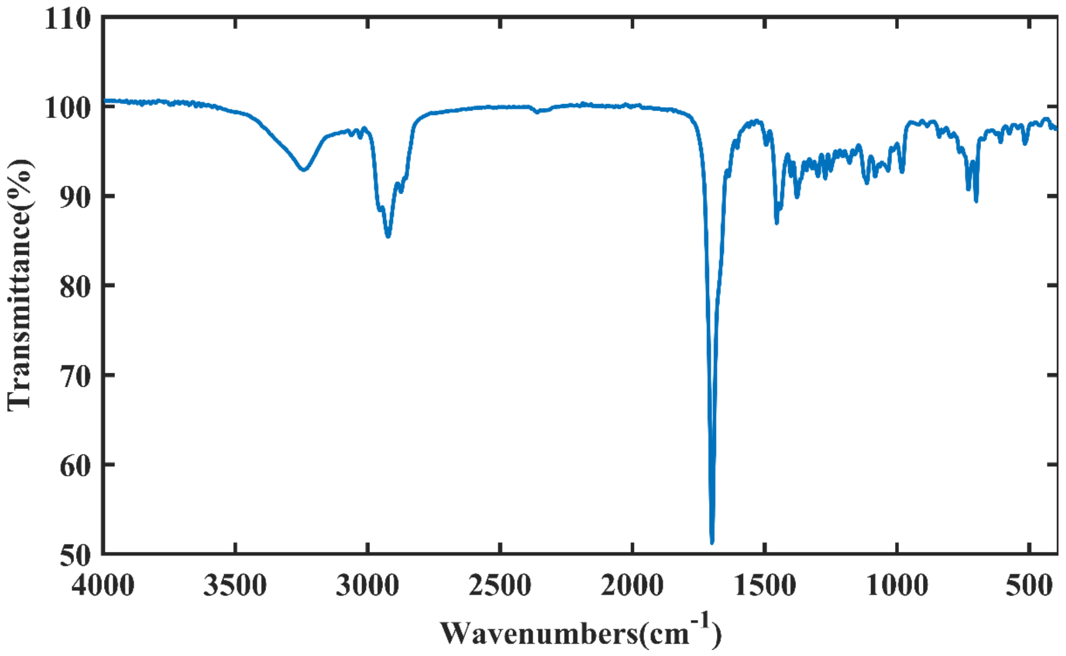

Figure S6. ^{1}H NMR (700 MHz, chloroform-*d*) spectrum of Malcirchalasin A (**7**).

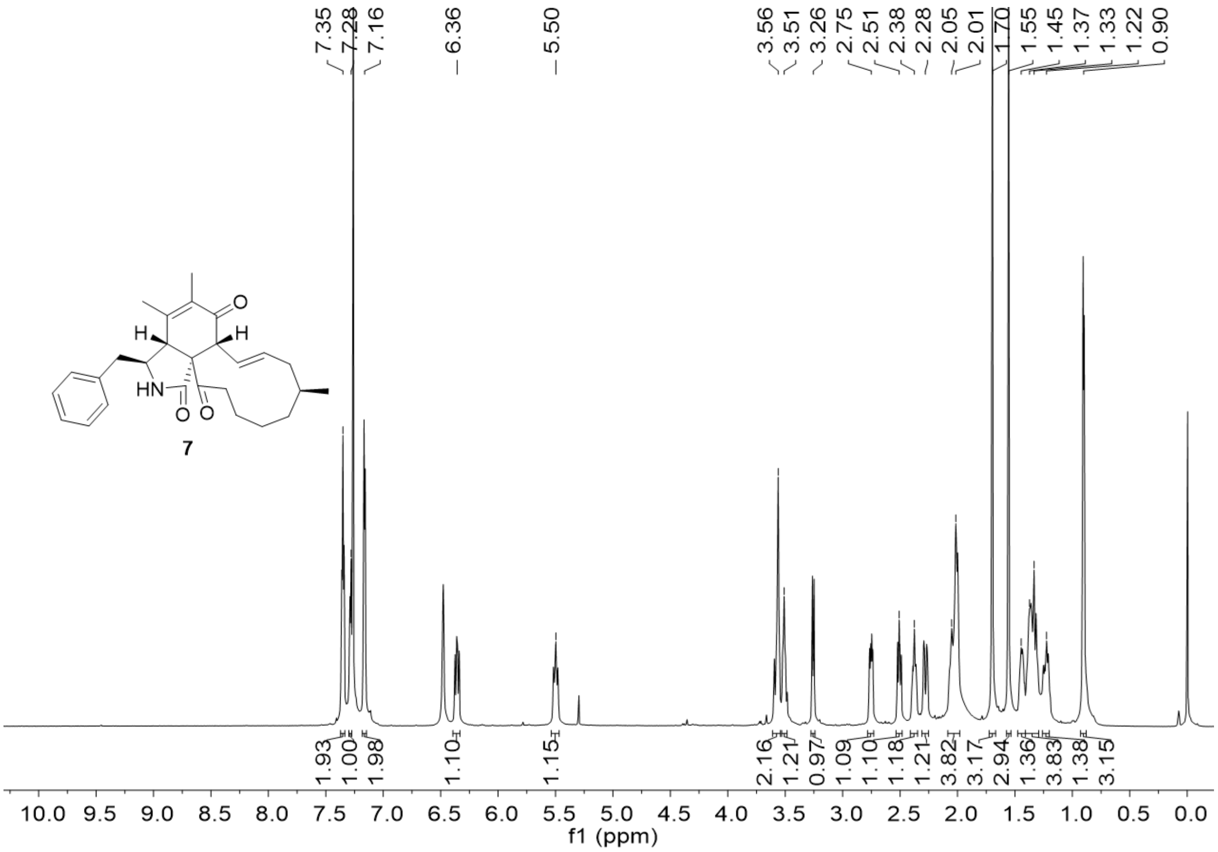

Figure S7. ^{13}C NMR (175 MHz, chloroform-*d*) spectrum of Malcirchalasin A (**7**).

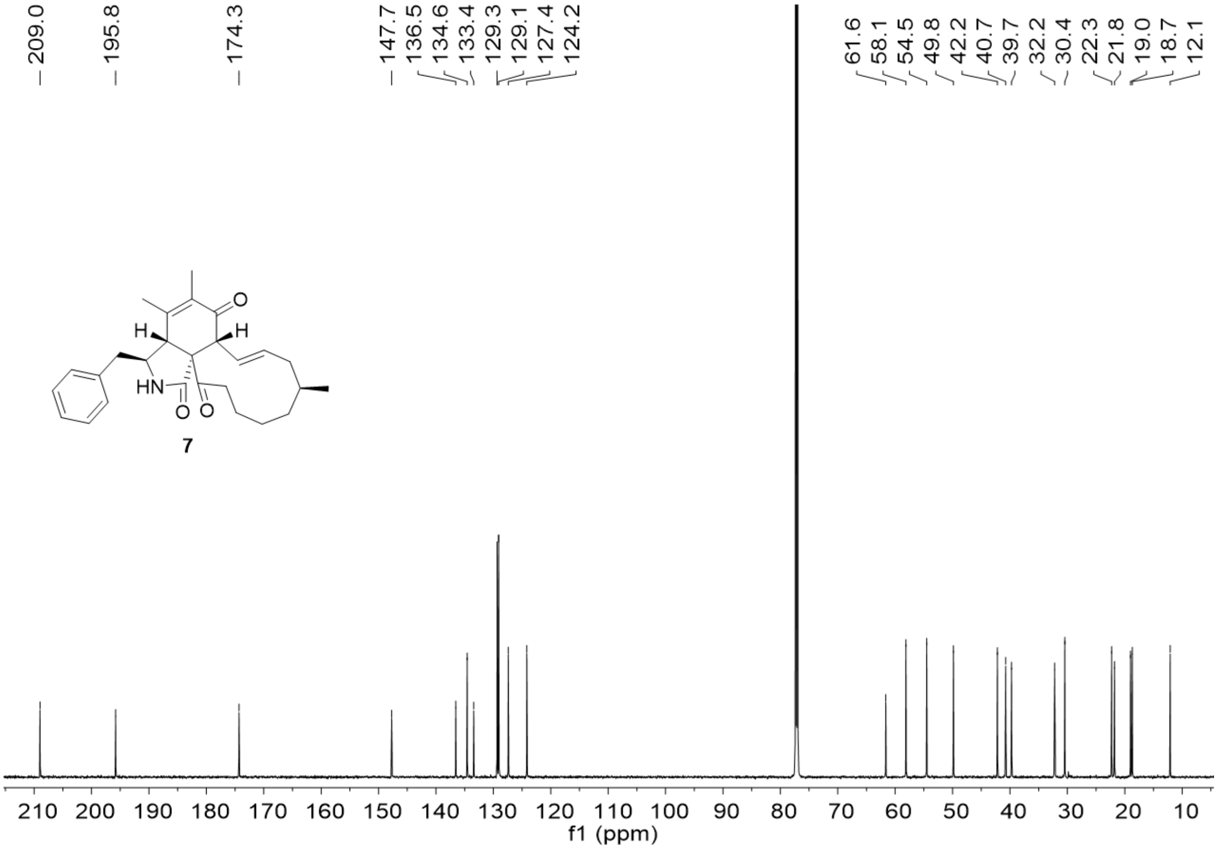

Figure S8. DEPT 135 (175 MHz, chloroform-*d*) spectrum of Malcirchalasin A (**7**).

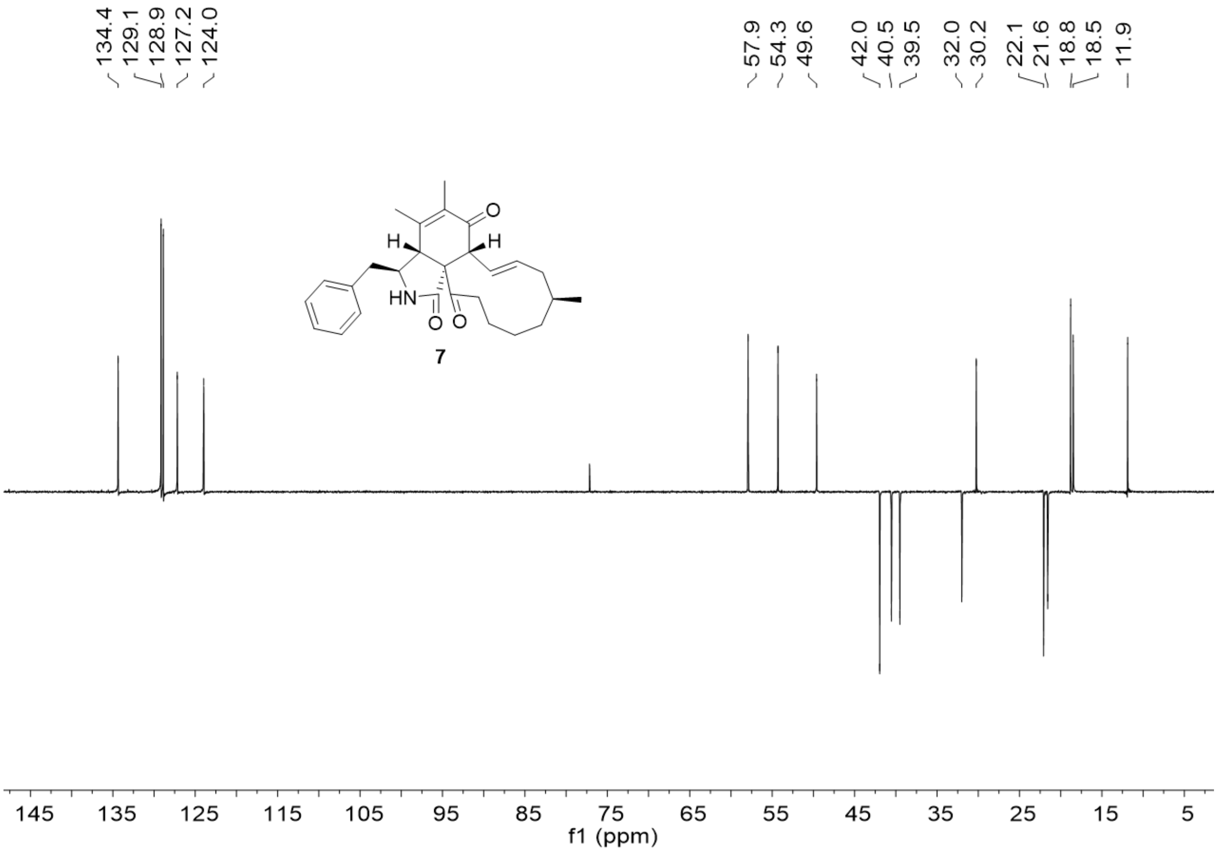

Figure S9. ^{1}H-^{1}H COSY spectrum of Malcirchalasin A (**7**).

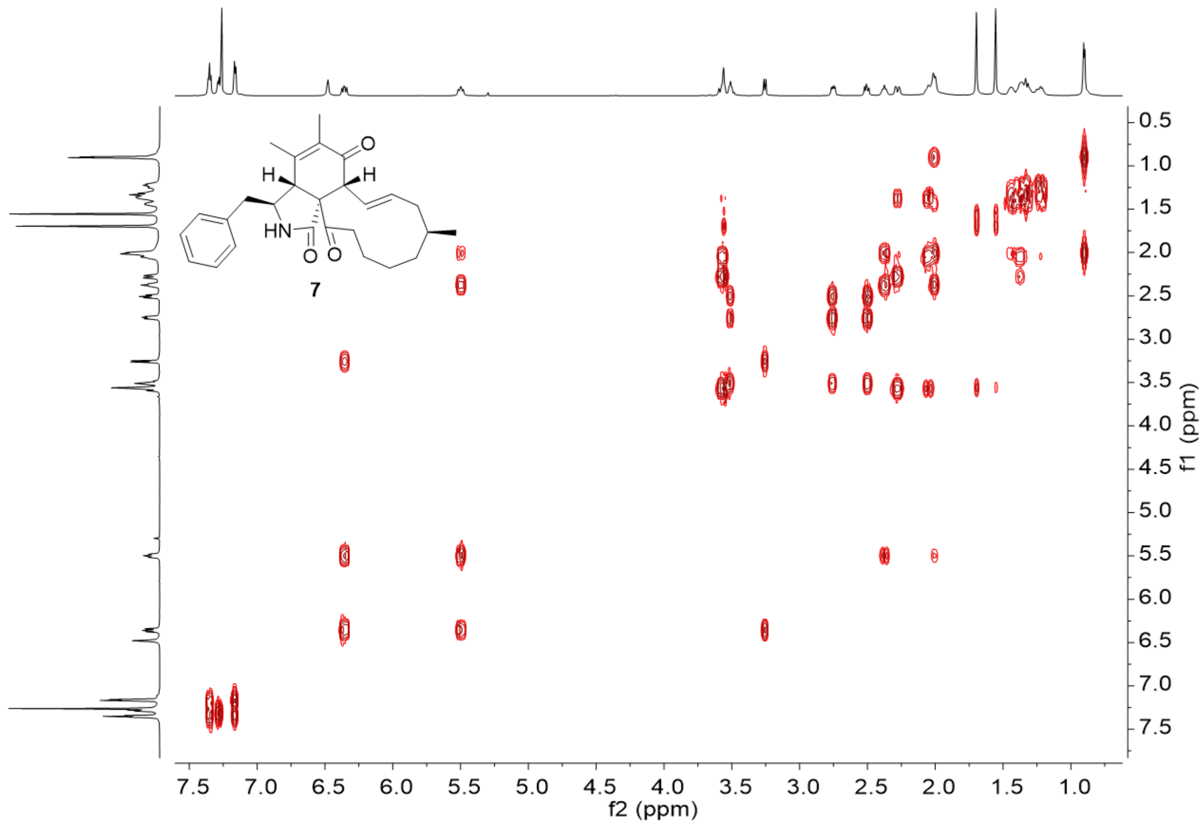

Figure S10. HSQC spectrum of Malcirchalasin A (**7**).

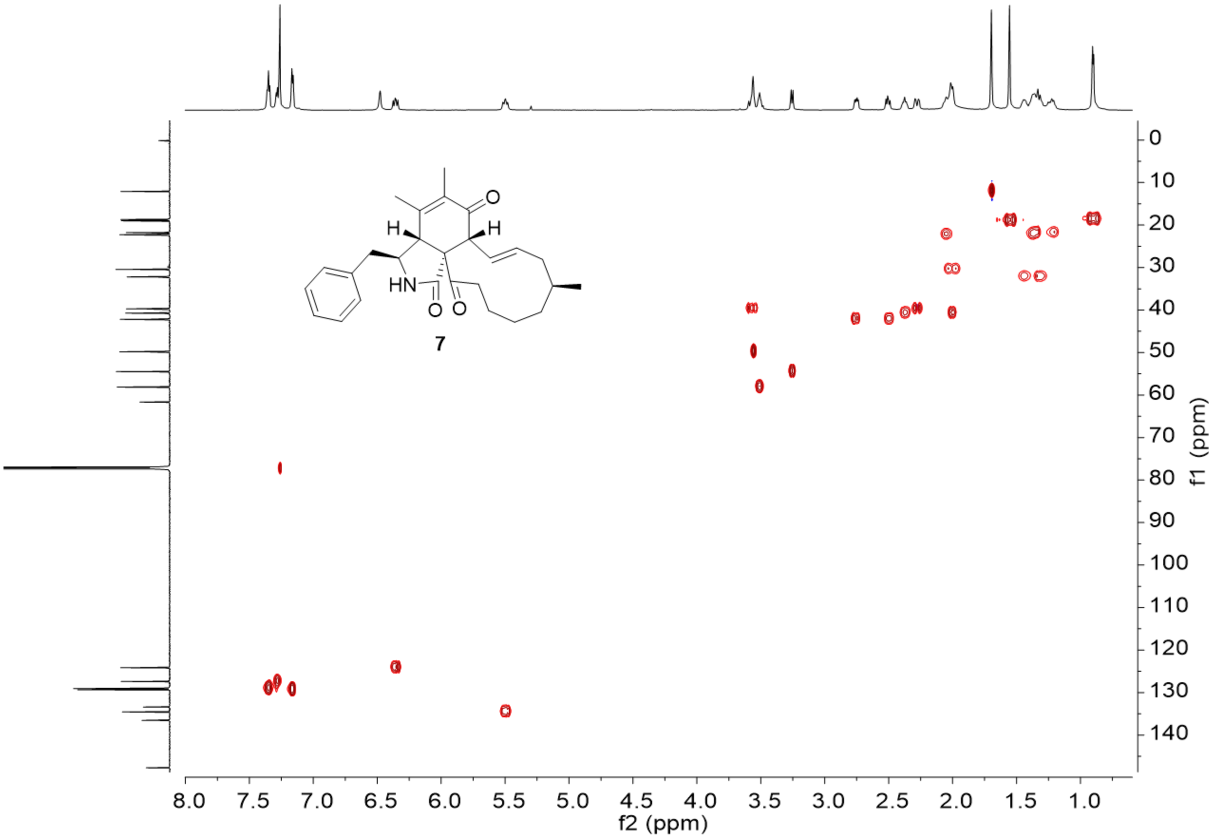

Figure S11. HMBC spectrum of Malcirchalasin A (**7**).

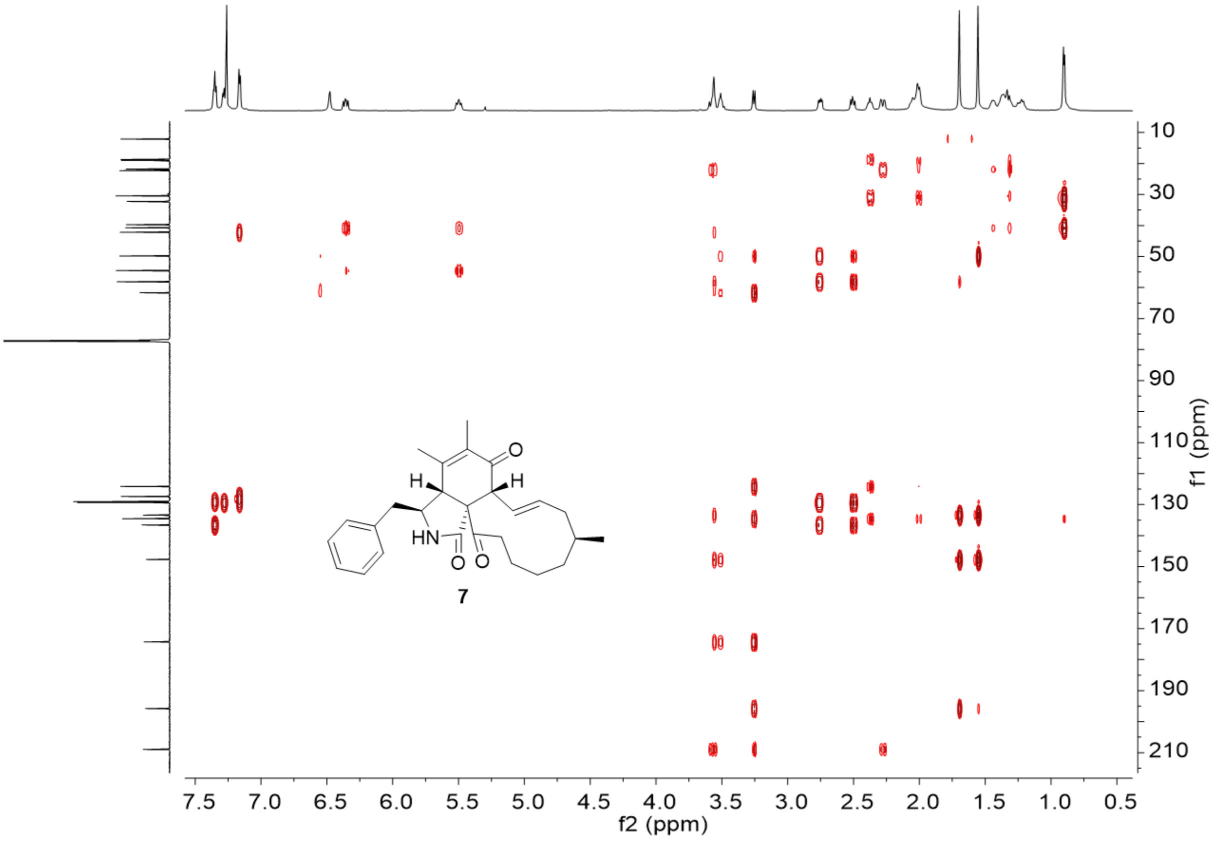

Figure S12. NOESY spectrum of Malcirchalasin A (**7**).

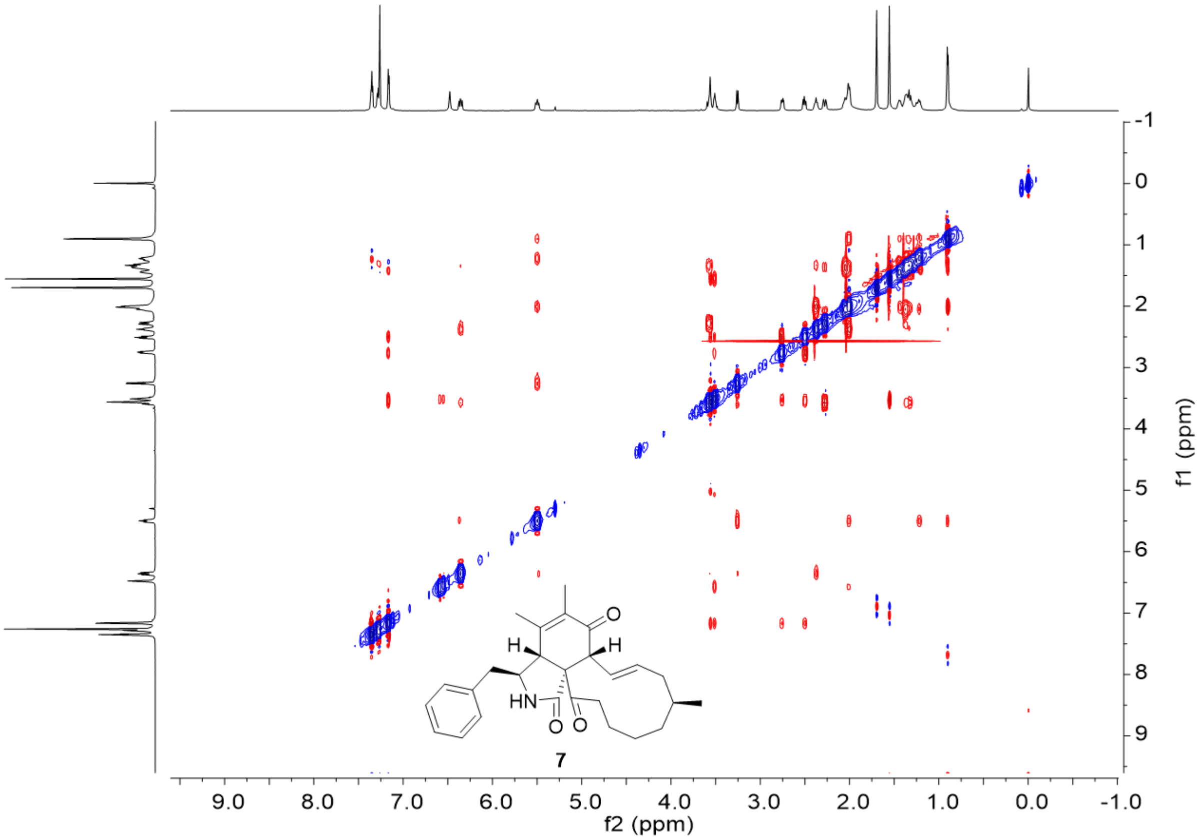

Figure S13. (+)-HRESIMS spectrum of Malcirchalasin B (**8**).

Mass Spectrum SmartFormula Report

Analysis Info

Analysis Name	D:\Data\MS\data\202408\yanghu_3036-5_pos_46_01_17825.d	Acquisition Date	8/16/2024 5:59:52 PM
Method	LC_Direct Infusion_pos_70-500mz.m	Operator	SCSIO
Sample Name	yanghu_3036-5_pos	Instrument	maXis 255552.00029
Comment			

Acquisition Parameter

Source Type	ESI	Ion Polarity	Positive	Set Nebulizer	0.4 Bar
Focus	Active	Set Capillary	4500 V	Set Dry Heater	180 °C
Scan Begin	70 m/z	Set End Plate Offset	-500 V	Set Dry Gas	4.0 l/min
Scan End	1500 m/z	Set Charging Voltage	0 V	Set Divert Valve	Waste
		Set Corona	0 nA	Set APCI Heater	0 °C

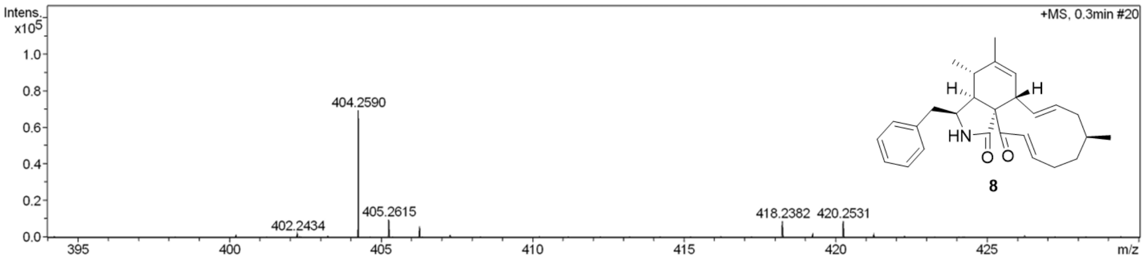

Meas. m/z	#	Ion Formula	Score	m/z	err [ppm]	err [mDa]	mSigma	rdb	e⁻ Conf	N-Rule
404.2590	1	C27H34NO2	100.00	404.2584	-1.5	-0.6	82.5	11.5	even	ok
807.5083	1	C54H67N2O4	100.00	807.5095	1.6	1.3	292.1	22.5	even	ok

Figure S14. IR spectrum of Malcirchalasin B (**8**).

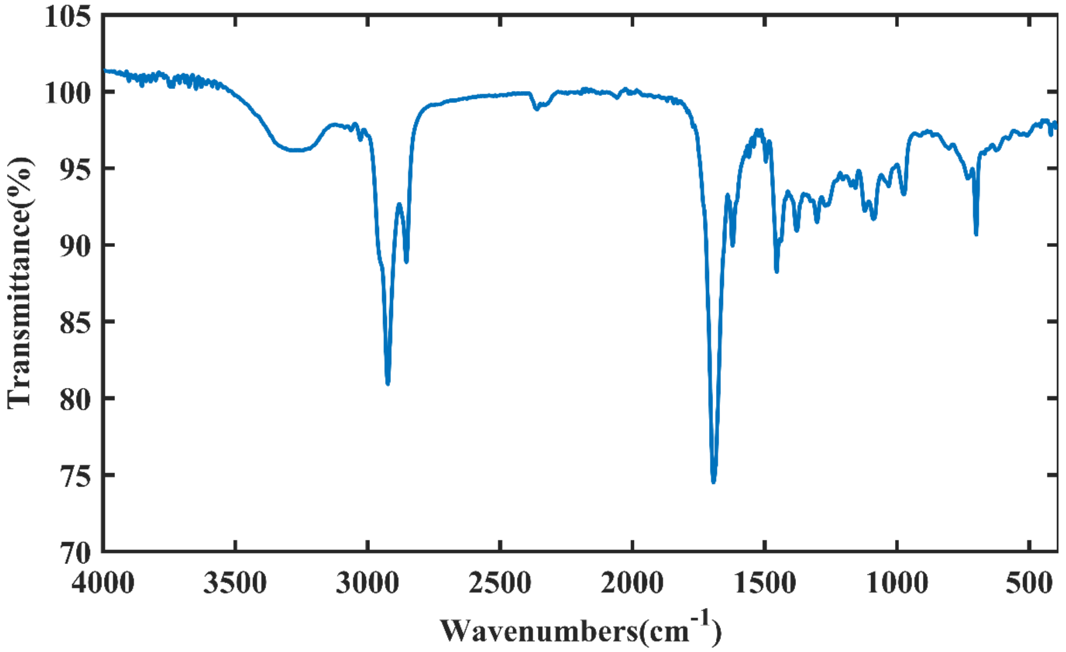

Figure S15. ^{1}H NMR (700 MHz, methanol-d_4) spectrum of Malcirchalasin B (**8**).

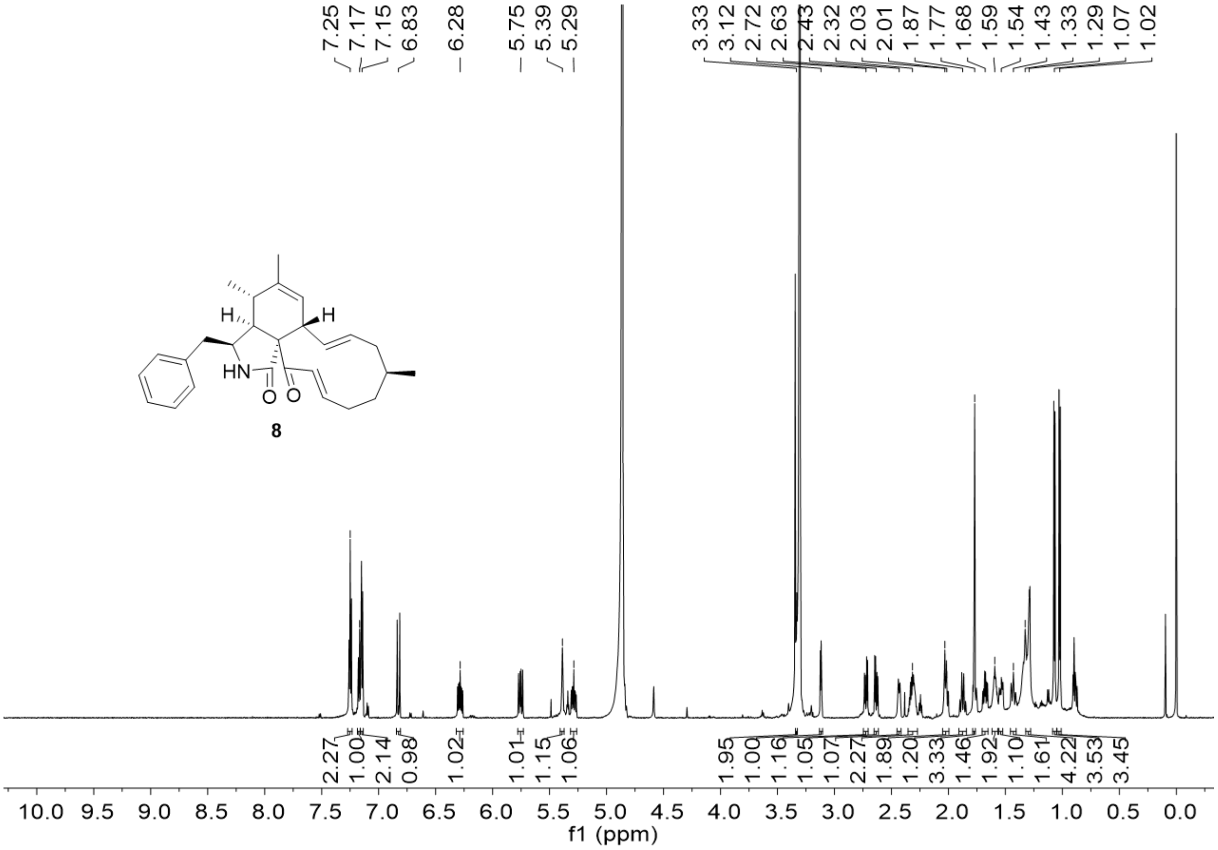

Figure S16. ^{13}C NMR (175 MHz, methanol-d_4) spectrum of Malcirchalasin B (**8**).

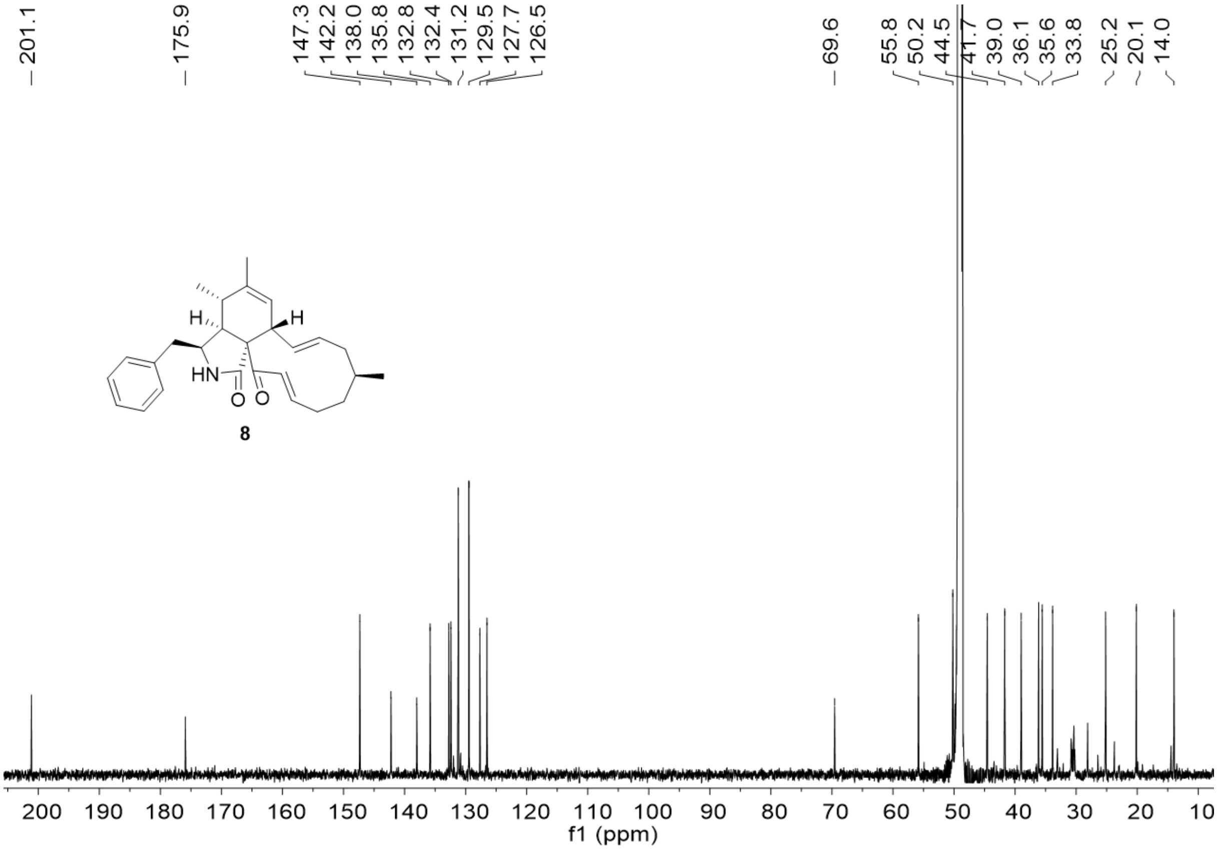

Figure S17. ^{1}H-^{1}H COSY spectrum of Malcirchalasin B (**8**).

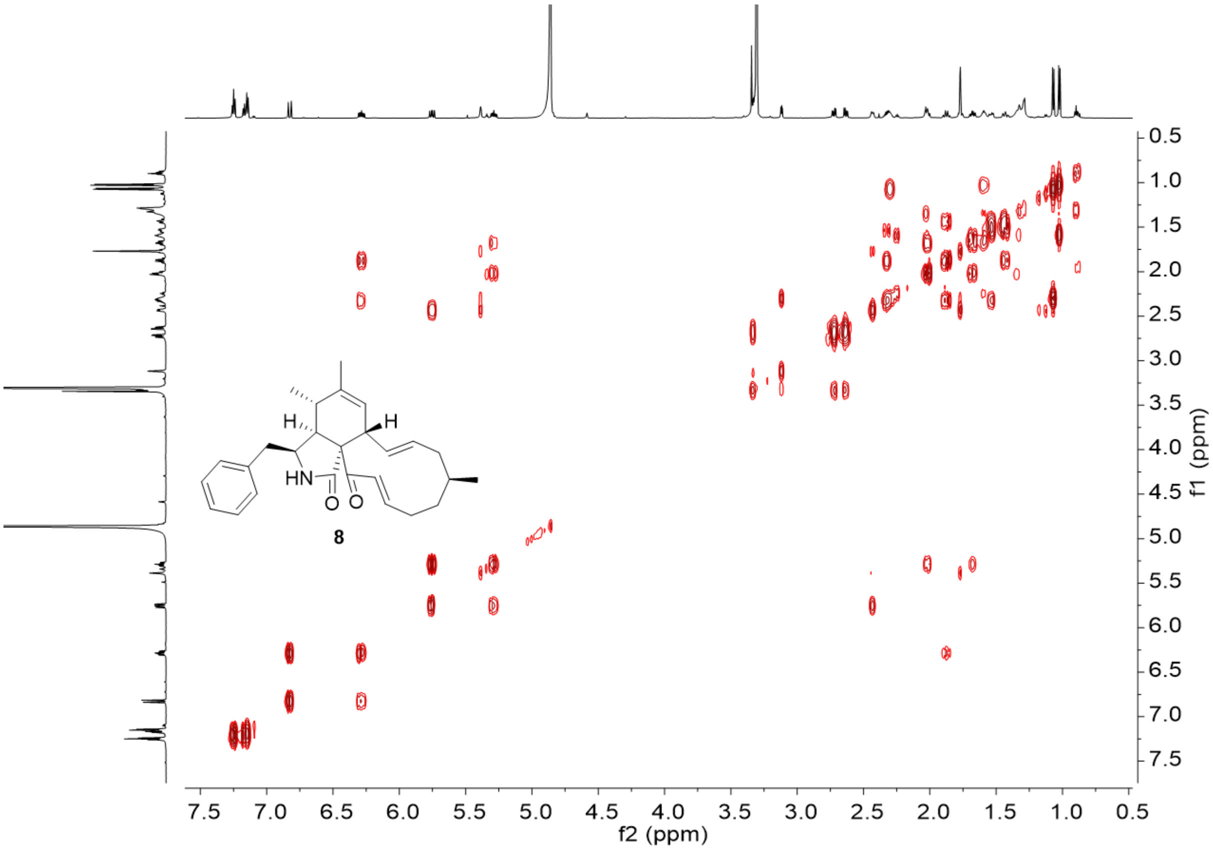

Figure S18. HSQC spectrum of Malcirchalasin B (**8**).

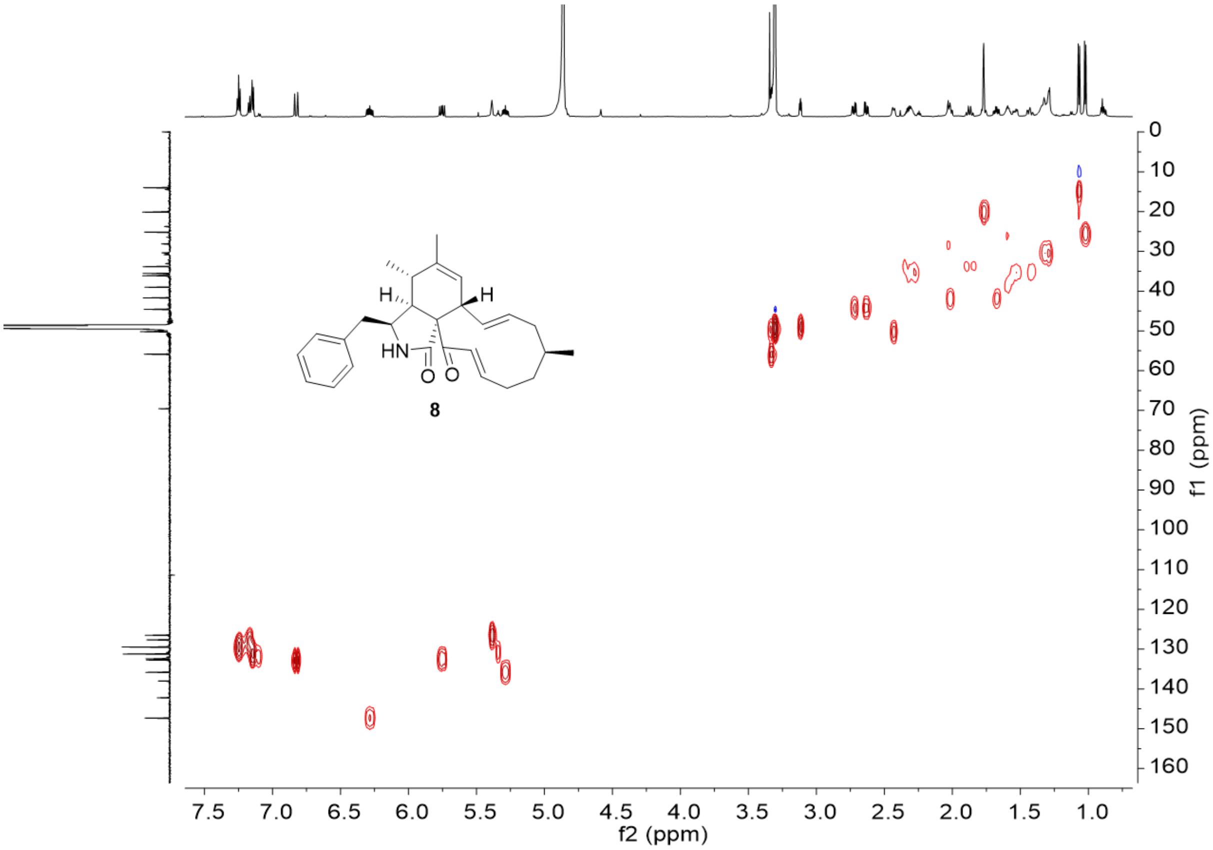

Figure S19. HMBC spectrum of Malcirchalasin B (**8**).

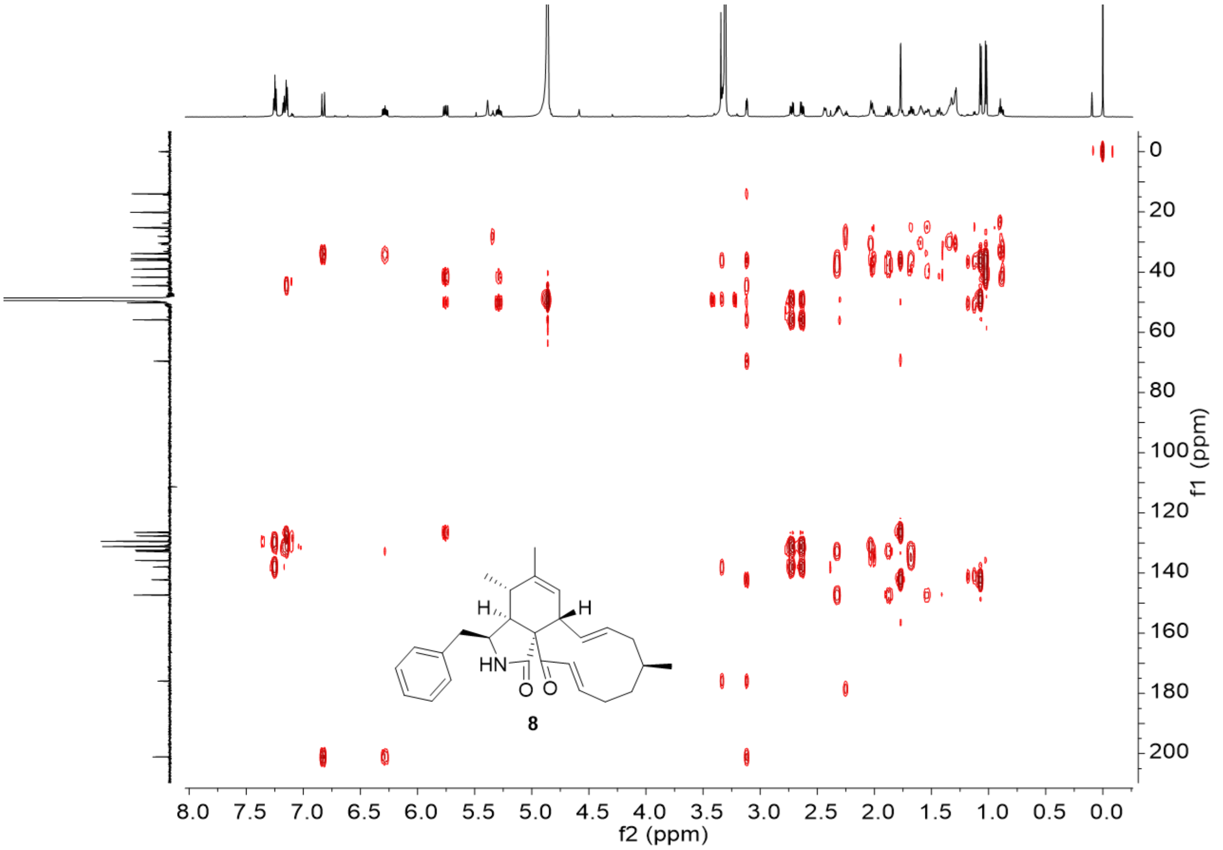

Figure S20. NOESY spectrum of Malcirchalasin B (**8**).

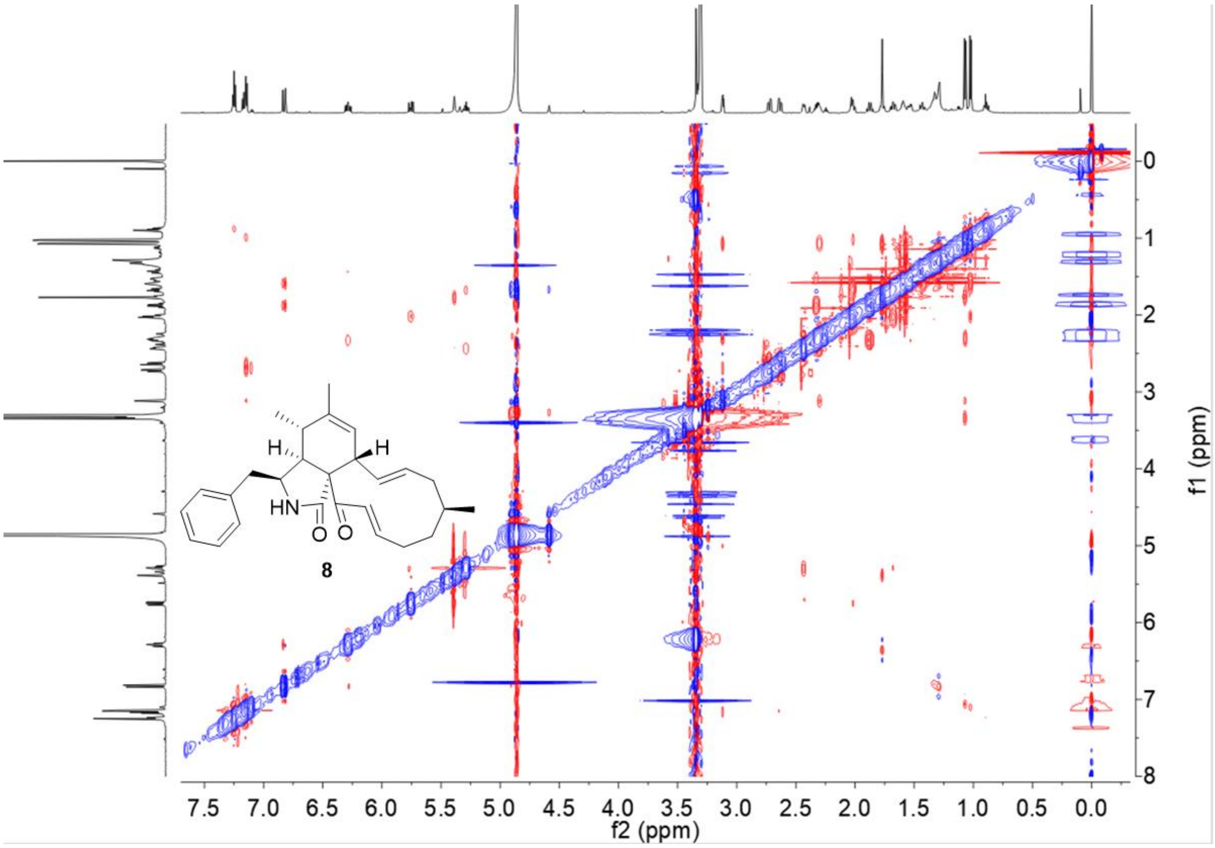

Figure S21. (+)-HRESIMS spectrum of Malcirchalasin C (**9**).

Mass Spectrum SmartFormula Report

Analysis Info

Analysis Name	D:\Data\MS\data\202408\yanghu_3036-4_pos_45_01_17824.d	Acquisition Date	8/16/2024 5:56:15 PM
Method	LC_Direct Infusion_pos_70-500mz.m	Operator	SCSIO
Sample Name	yanghu_3036-4_pos	Instrument	maXis 255552.00029
Comment			

Acquisition Parameter

Source Type	ESI	Ion Polarity	Positive	Set Nebulizer	0.4 Bar
Focus	Active	Set Capillary	4500 V	Set Dry Heater	180 °C
Scan Begin	70 m/z	Set End Plate Offset	-500 V	Set Dry Gas	4.0 l/min
Scan End	1500 m/z	Set Charging Voltage	0 V	Set Divert Valve	Waste
		Set Corona	0 nA	Set APCI Heater	0 °C

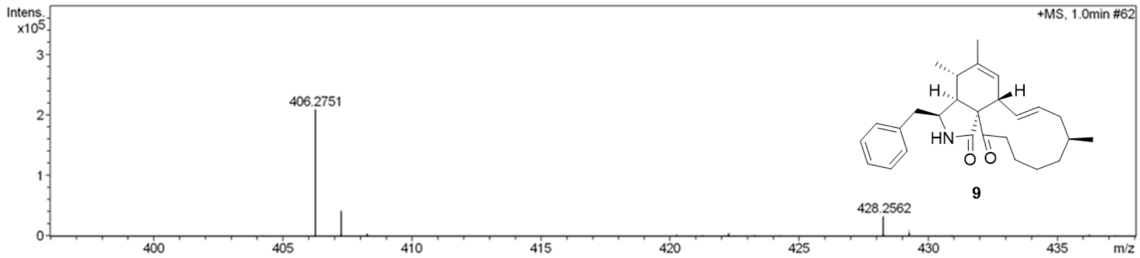

Meas. m/z	#	Ion Formula	Score	m/z	err [ppm]	err [mDa]	mSigma	rdb	e⁻ Conf	N-Rule
406.2751	1	C27H36NO2	100.00	406.2741	2.5	1.0	52.3	10.5	even	ok
428.2562	1	C27H35NNaO2	100.00	428.2560	-0.5	-0.2	68.0	10.5	even	ok
811.5430	1	C54H71N2O4	100.00	811.5408	-2.7	-2.2	37.3	20.5	even	ok
833.5246	1	C54H70N2NaO4	100.00	833.5228	-2.2	-1.8	61.7	20.5	even	ok

Figure S22. IR spectrum of Malcirchalasin C (**9**).

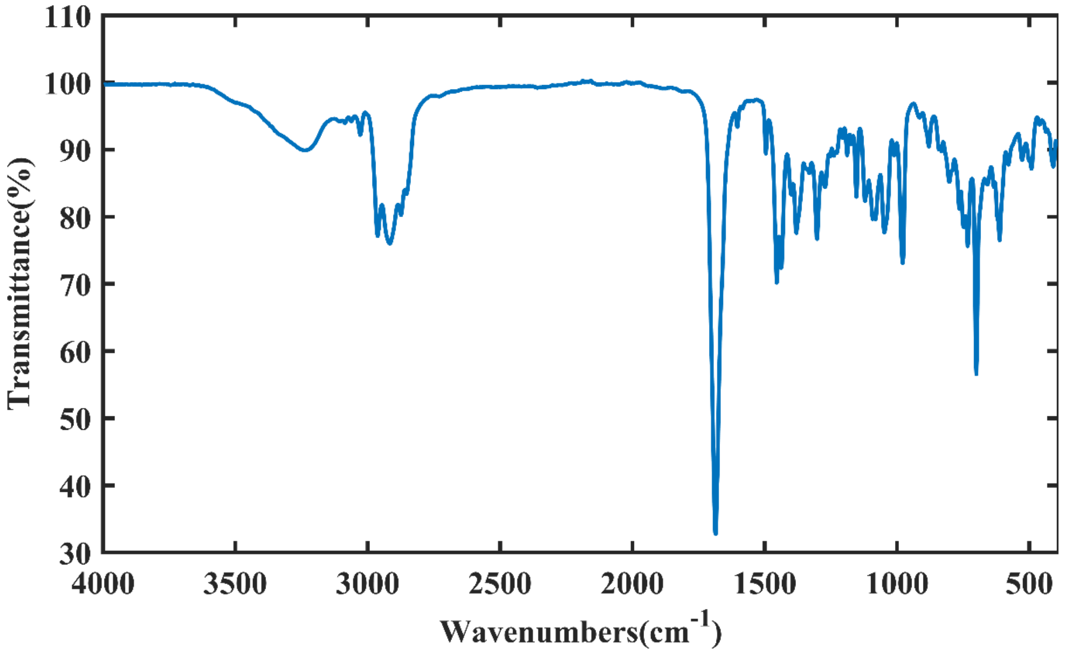

Figure S23. ^{1}H NMR (700 MHz, methanol-d_4) spectrum of Malcirchalasin C (**9**).

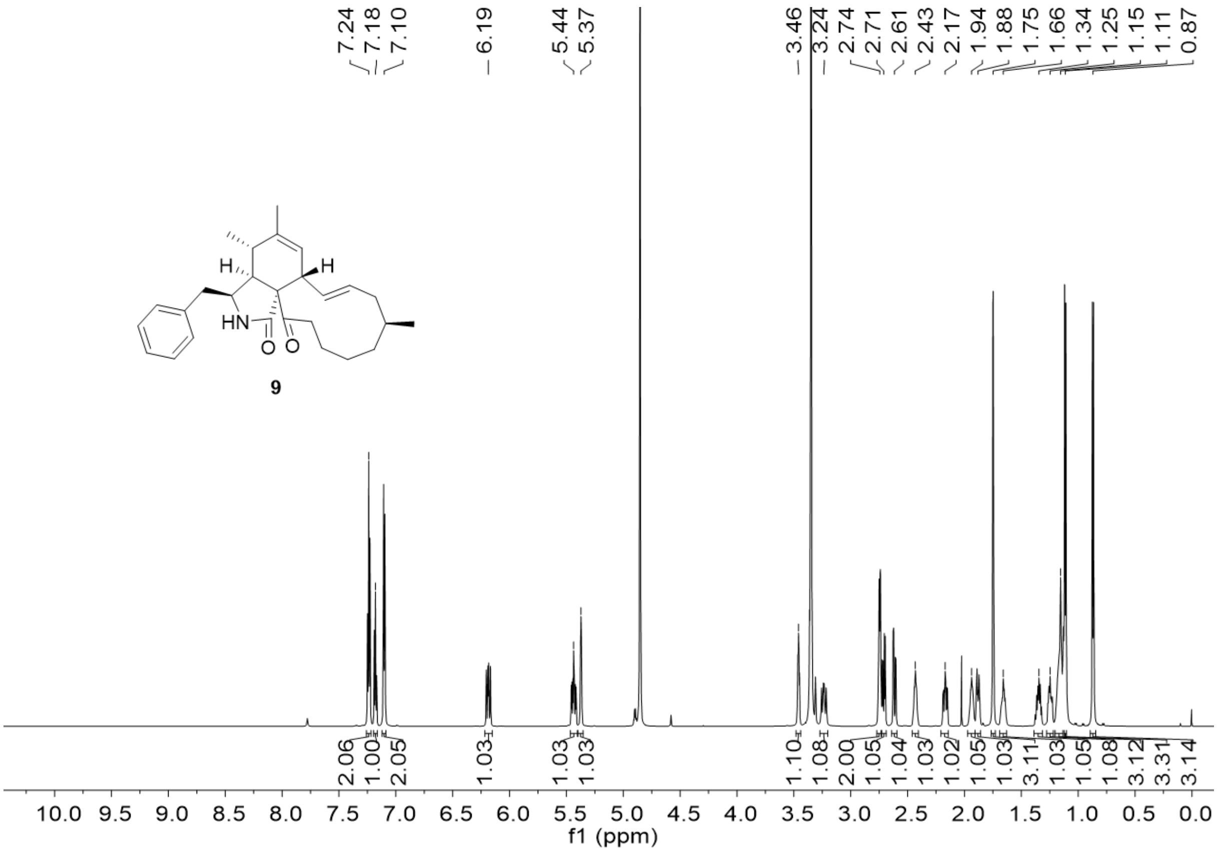

Figure S24. ^{13}C NMR (175 MHz, methanol-d_4) spectrum of Malcirchalasin C (**9**).

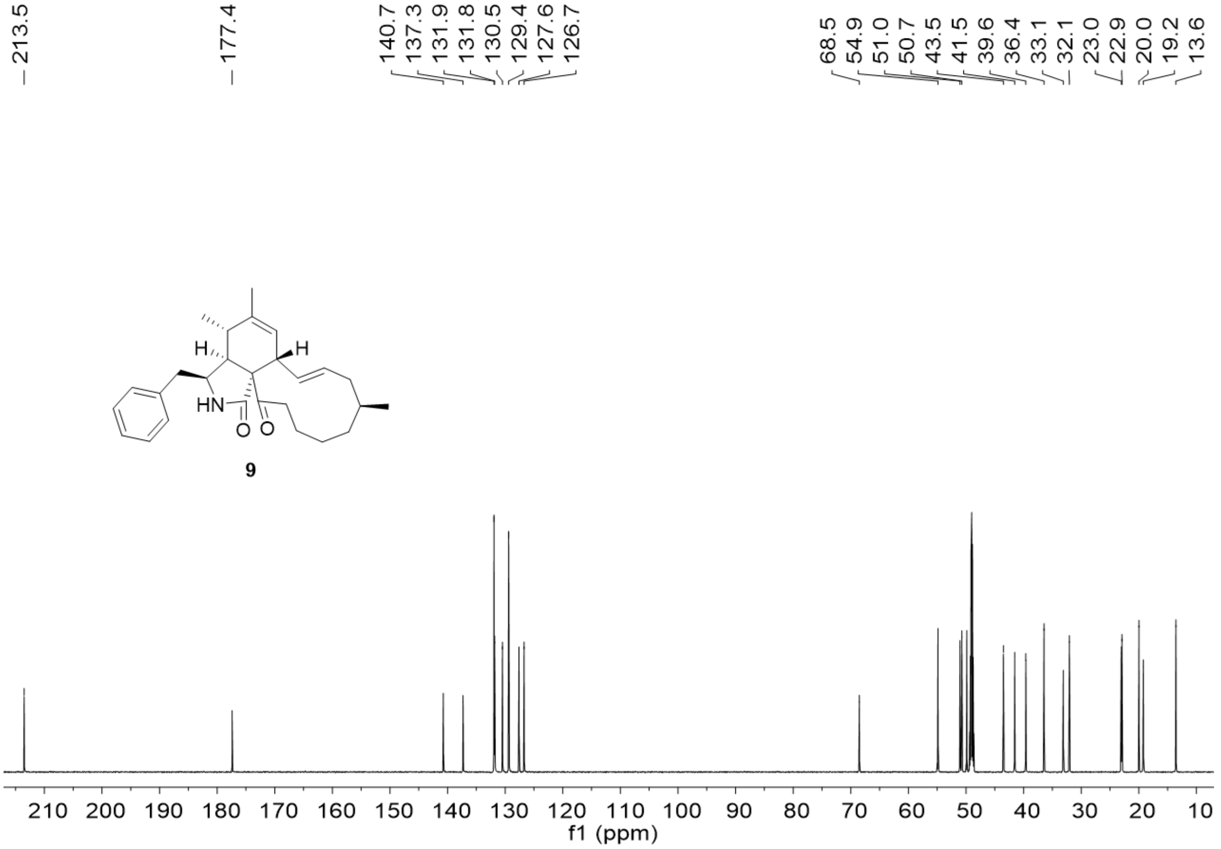

Figure S25. ^{1}H-^{1}H COSY spectrum of Malcirchalasin C (**9**).

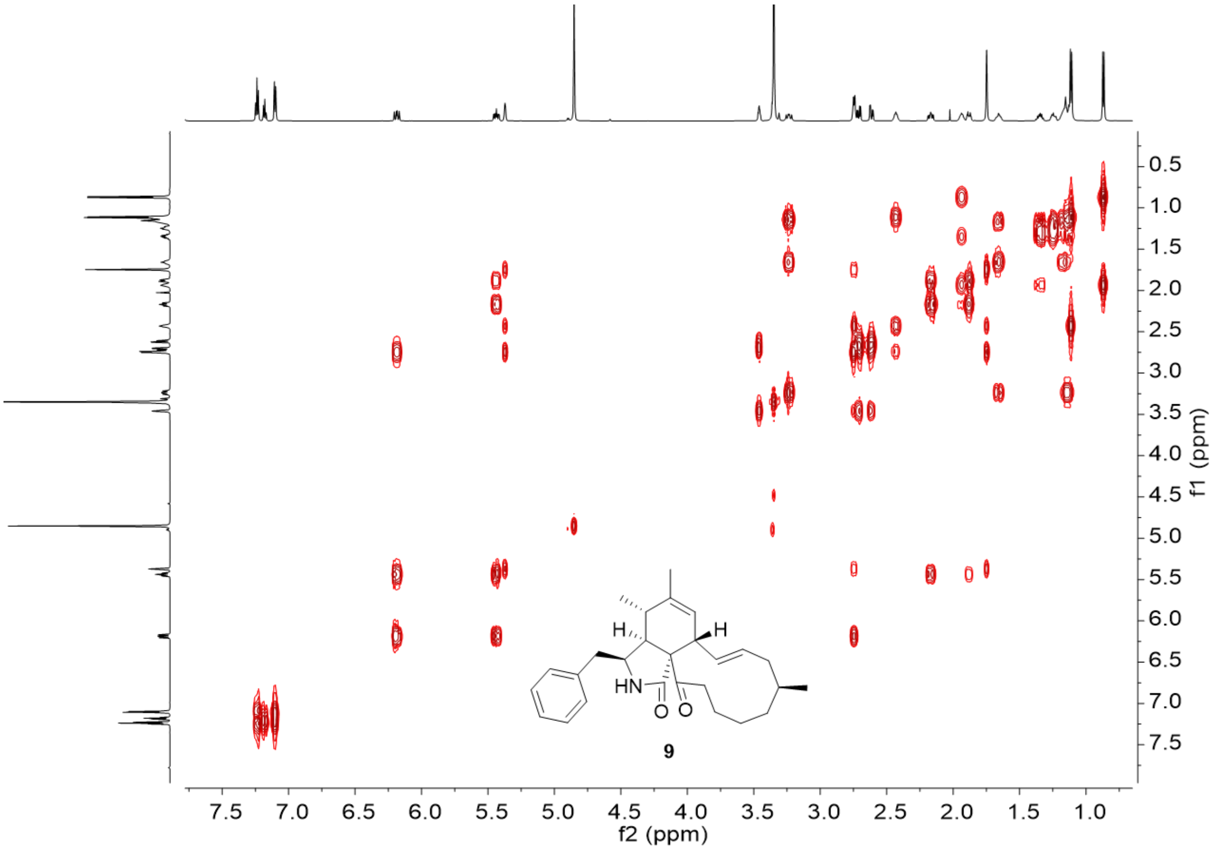

Figure S26. HSQC spectrum of Malcirchalasin C (**9**).

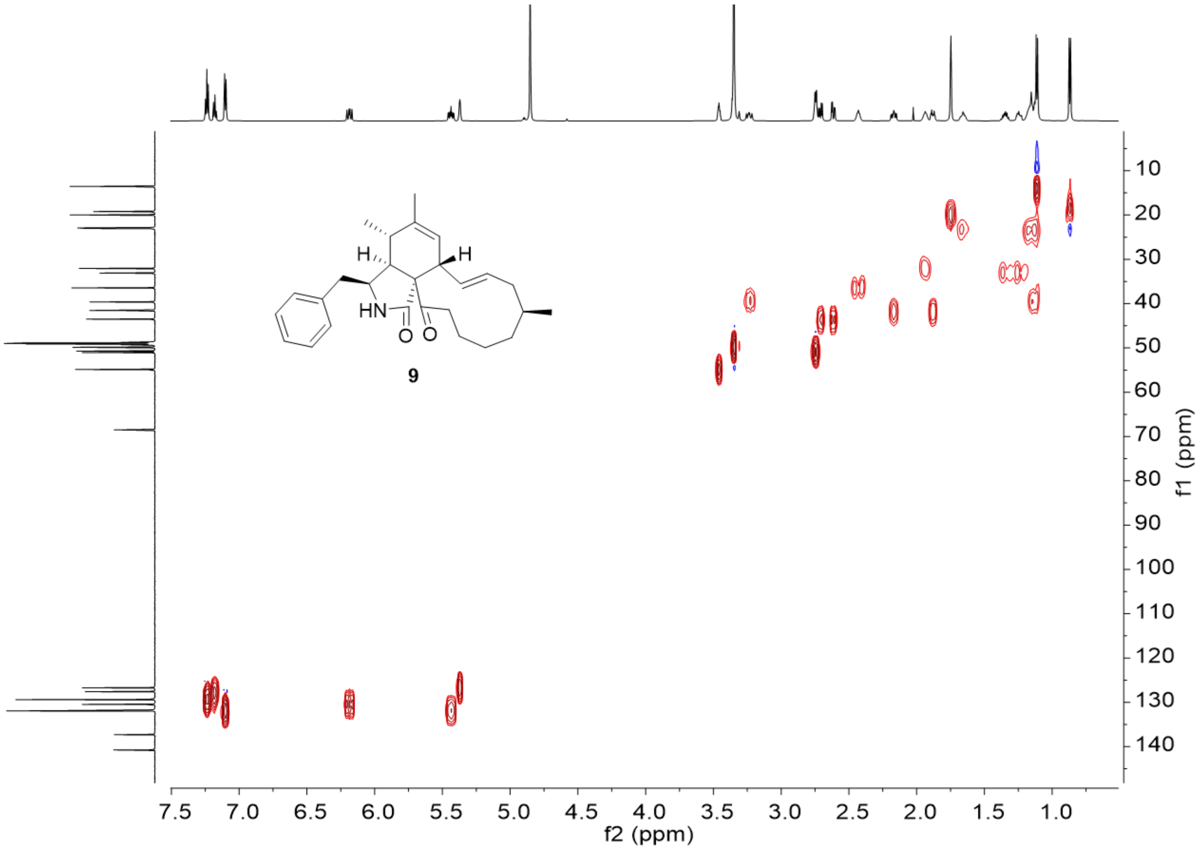

Figure S27. HMBC spectrum of Malcirchalasin C (**9**).

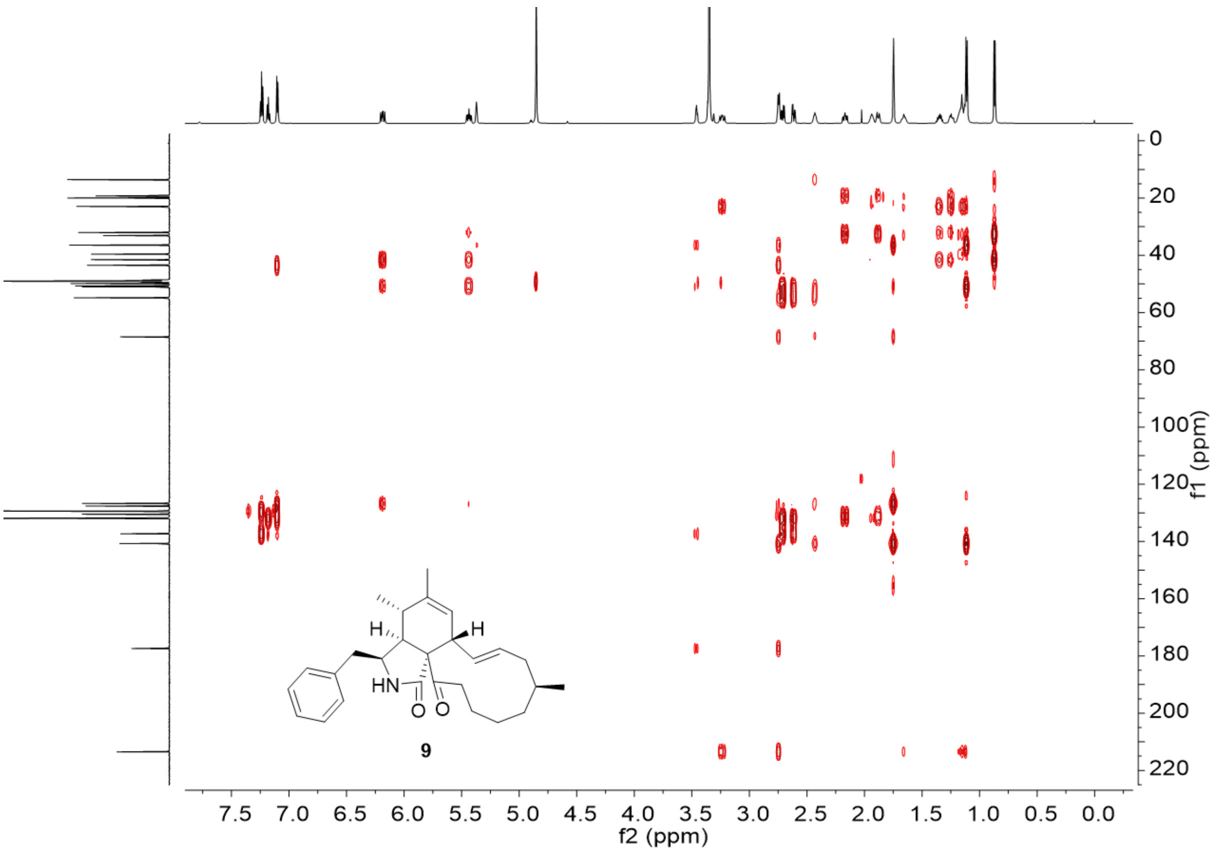

Figure S28. NOESY spectrum of Malcirchalasin C (**9**).

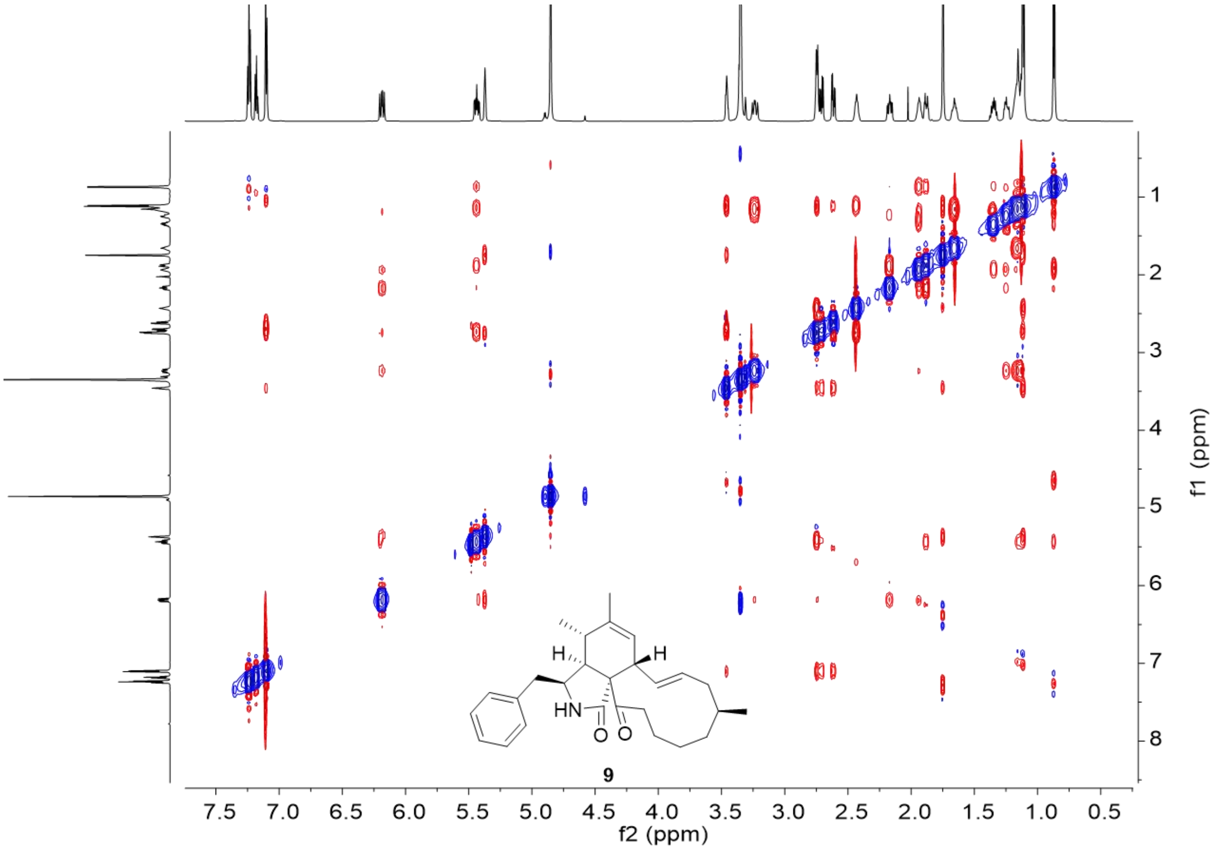

Figure S29. The optimized conformers and equilibrium populations of **7**.

(3*S*, 4*R*, 8*R*, 9*R*, 16*S*)-**7**

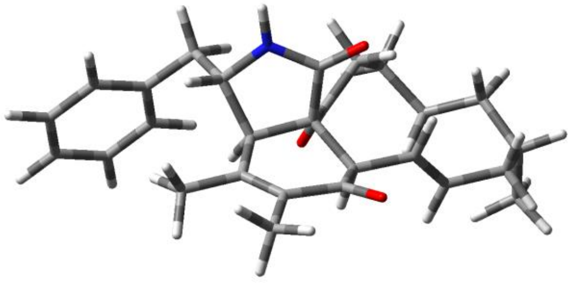

Conf.1 (53.35%)

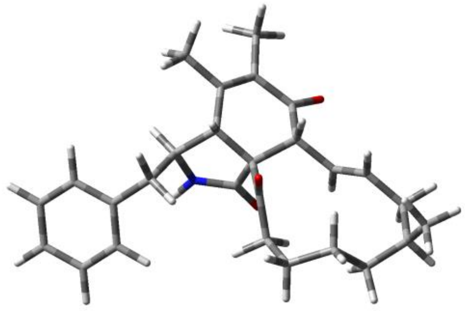

Conf.2 (10.19%)

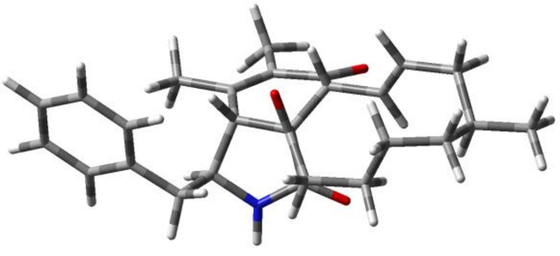

Conf.3 (11.45%)

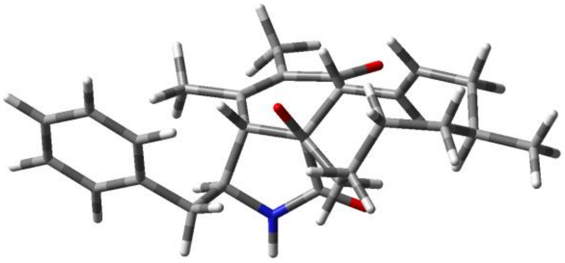

Conf.4 (6.42%)

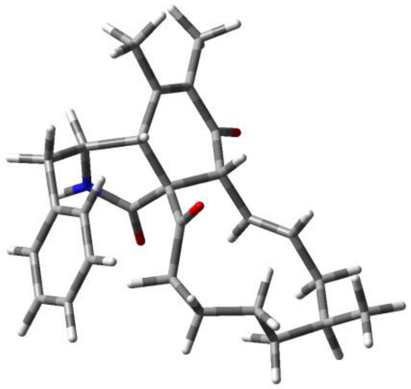

Conf.5 (18.58%)

Figure S30. The optimized conformers and equilibrium populations of **8**.

(3*S*, 4*S*, 5*S*, 8*S*, 9*S*, 16*S*)-**8**

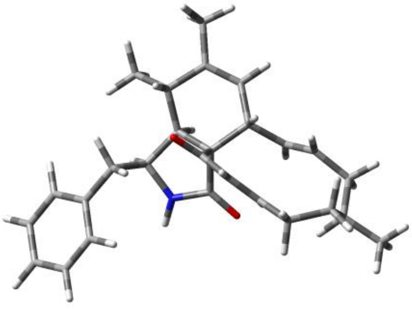

Conf.1 (68.22%)

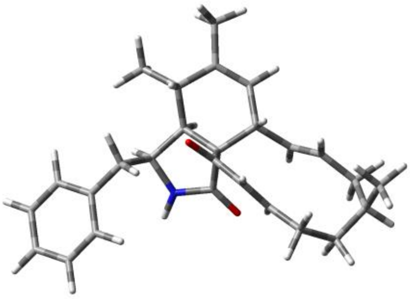

Conf.2 (1.09%)

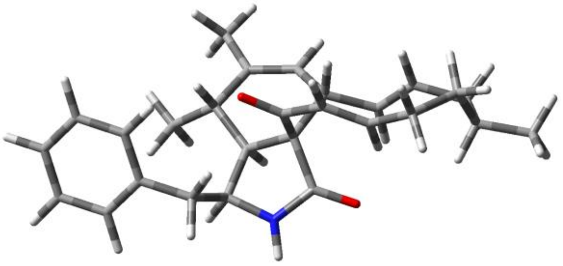

Conf.3 (30.67%)

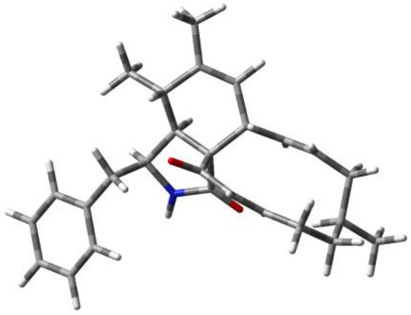

Conf.4 (0.02%)

(3*S*, 4*R*, 5*S*, 8*S*, 9*S*, 16*S*)-**8**

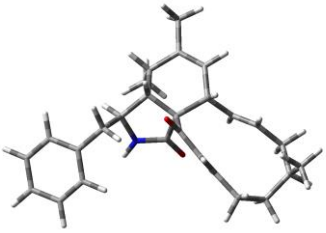

Conf.1 (0.09%)

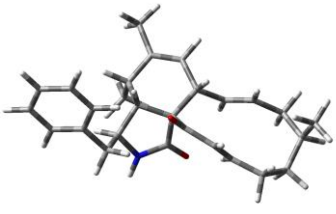

Conf.2 (0.18%)

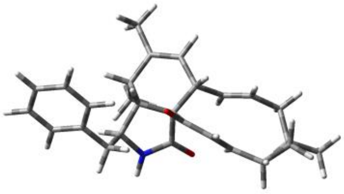

Conf.3 (1.05%)

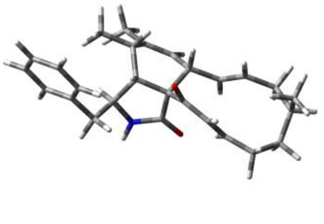

Conf.4 (26.85%)

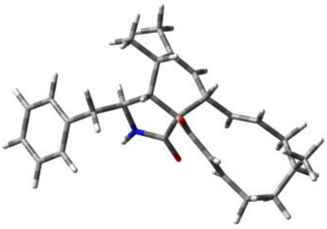

Conf.5 (30.33%)

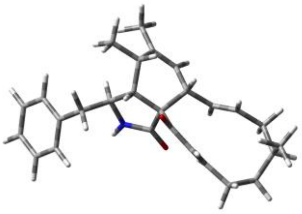

Conf.6 (30.33%)

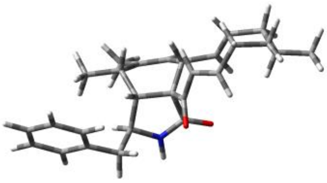

Conf.7 (8.33%)

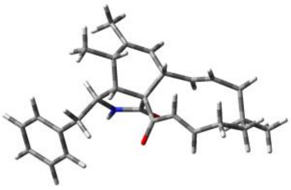

Conf.8 (2.84%)

Figure S31. Molecular network of secondary metabolites in extracts of *Malbranchea circinata* SDU050.

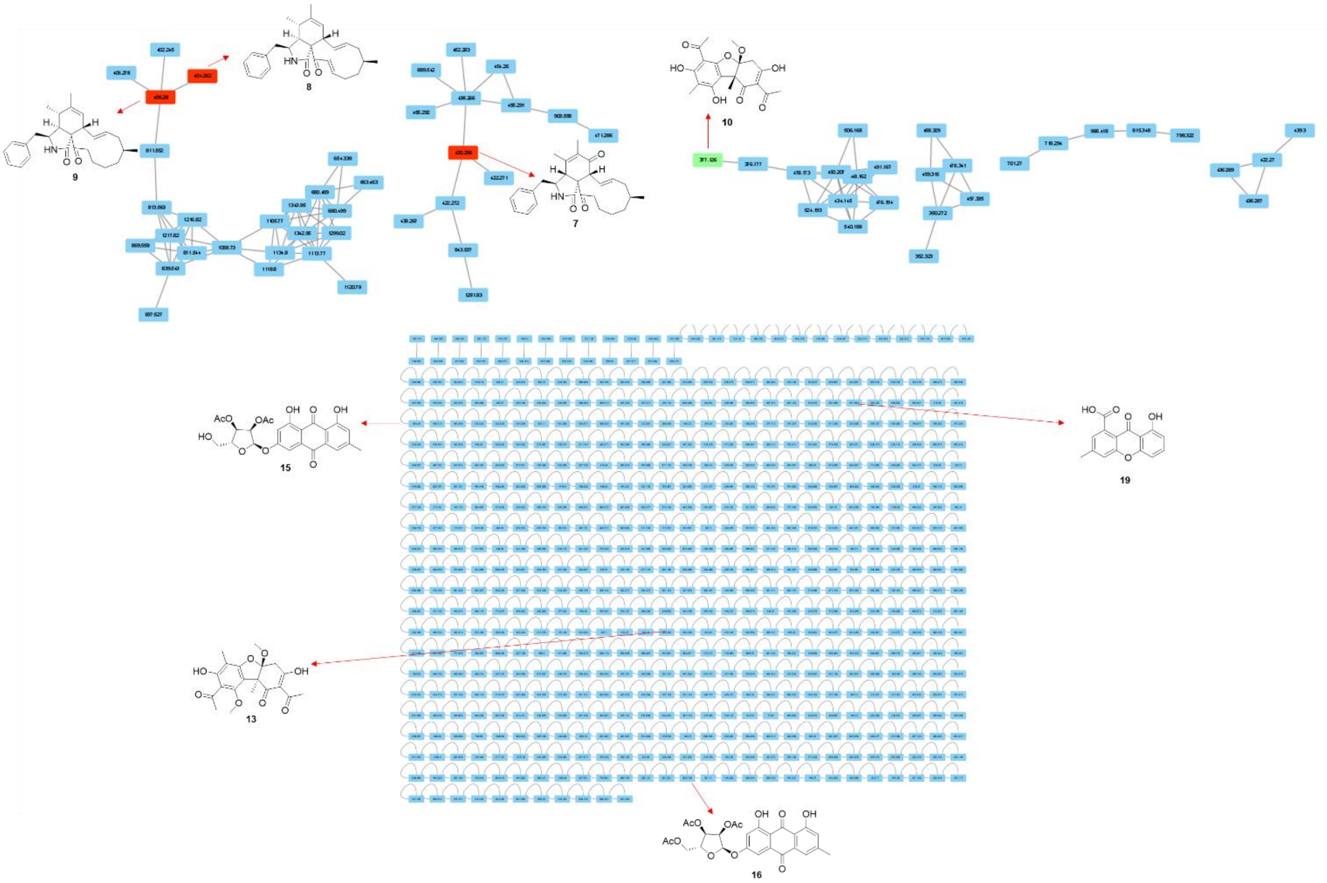

Supplement: Supplementary file 1 [file marinedrugs-23-00050-s001.zip › marinedrugs-3427193-supplementary.pdf]
